# Regulated somatic hypermutation enhances antibody affinity maturation

**DOI:** 10.1038/s41586-025-08728-2

**Published:** 2025-03-19

**Authors:** Julia Merkenschlager, Andrew G. T. Pyo, Gabriela S. Silva Santos, Dennis Schaefer-Babajew, Melissa Cipolla, Harald Hartweger, Alexander D. Gitlin, Ned S. Wingreen, Michel C. Nussenzweig

**Affiliations:** 1 Laboratory of Molecular Immunology, The Rockefeller University, New York, NY USA; 2 Laboratory of Lymphocyte Collaboration and Competition, Harvard Medical School, Boston, MA USA; 3 Department of Physics, Princeton University, Princeton, NJ USA; 4 Immunology Program, Memorial Sloan Kettering Cancer Center, New York, NY USA; 5 Department of Pathology and Laboratory Medicine, Memorial Sloan Kettering Cancer Center, New York, NY USA; 6 Department of Molecular Biology, Princeton University, Princeton, NJ USA; 7 Lewis-Sigler Institute for Integrative Genomics, Princeton University, Princeton, NJ USA; 8 Howard Hughes Medical Institute, The Rockefeller University, New York, NY USA

**Keywords:** Cell vaccines, Immunology, Adaptive immunity, Autoimmunity

## Abstract

Germinal centres are specialized microenvironments where B cells undergo affinity maturation. B cells expressing antibodies whose affinity is improved by somatic hypermutation are selected for expansion by limiting numbers of T follicular helper cells. Cell division is accompanied by mutation of the immunoglobulin genes, at what is believed to be a fixed rate of around 1 × 10^−3^ per base pair per cell division^1^. As mutagenesis is random, the probability of acquiring deleterious mutations outweighs the probability of acquiring affinity-enhancing mutations. This effect might be heightened, and even become counterproductive, in B cells that express high-affinity antibodies and undergo the greatest number of cell divisions^2^. Here we experimentally examine a theoretical model that explains how affinity maturation could be optimized by varying the rate of somatic hypermutation such that cells that express higher-affinity antibodies divide more but mutate less per division. Data obtained from mice immunized with SARS-CoV-2 vaccines or a model antigen align with the theoretical model and show that cells producing high-affinity antibodies shorten the G0/G1 phases of the cell cycle and reduce their mutation rates. We propose that these mechanisms safeguard high-affinity B cell lineages and enhance the outcomes of antibody affinity maturation.

## Abstract (web summary)

Germinal centre B cells modify their mutation rate to preserve high-affinity receptors, thereby safeguarding high-affinity B cell lineages and enhancing the outcomes of antibody affinity maturation.

## Main

Within germinal centres (GCs), B cells cycle between two zones: the dark zone (DZ) and the light zone (LZ)^3–5^. In the LZ, B cells compete for limited T follicular helper (T_FH_) cell help, which selects B cells based on antibody affinity for DZ re-entry. The magnitude of T_FH_ cell help determines the level of c-Myc, which regulates both the speed and number of B cell divisions made in the DZ^6,7^, wherein B cells also undergo somatic hypermutation (SHM) to diversify their antibody genes^3,8,9^. Since nuclear membrane breakdown during mitosis is essential for DNA mutagenesis, SHM and cell division are closely linked^10–12^. Current understanding suggests that SHM continues at a constant rate per division, so that the highest-affinity B cells, which divide more often, also accumulate more mutations than their lower affinity counterparts^13^. However, because SHM is random, with most mutations decreasing affinity rather than enhancing it^3,8,9,14^, B cells dividing the most might disadvantage their progeny by acquiring more mutations. We propose and experimentally test a model in which the survival of high-affinity B cell lineages is enhanced by modulating the rate of SHM per division, allowing B cells with the highest-affinity antibodies to undergo mutation-free proliferative bursts^15,16^.

Despite the relatively low probability that any one mutation enhances affinity, GC responses can produce 100-fold increases in serum antibody affinity within a short period of time^17^. This phenomenon led us to investigate how cycles of mutation and selection might be optimized in the GC^15^. Herein, we sought to determine whether affinity maturation is inherently wasteful with diminishing returns for higher-affinity cells, or whether it includes mechanisms to protect these cells from accumulating affinity-reducing mutations.

**Fig. 1:**
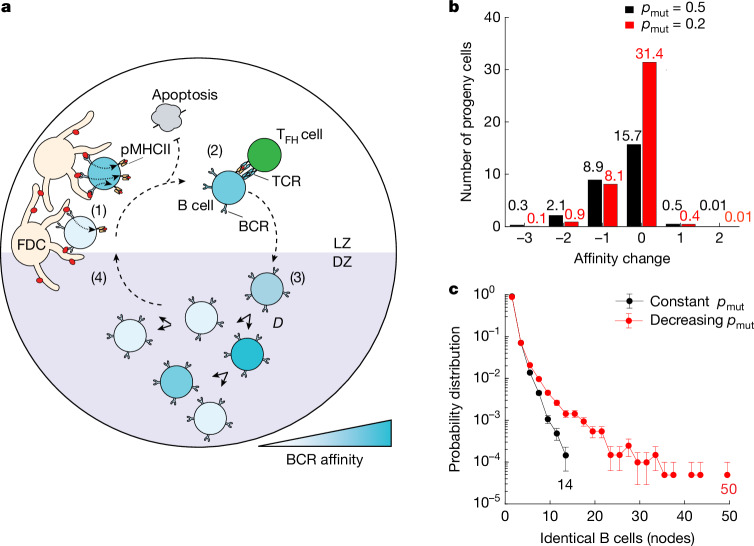
Agent-based model of the GC reaction. **a**, Diagrammatic representation of key processes involved in GC reactions captured by the agent-based model (Supplementary Table 1; Methods). TCR, T cell receptor; pMHCII, peptide major histocompatibility complex II. **b**, Comparison of the effect of different mutation rates on B cell progeny affinities. Graph shows the number of progeny cells versus the net change in affinity produced after six consecutive divisions in the DZ, assuming a constant (*p*_mut_ = 0.5 (black)) or a lower (*p*_mut_ = 0.2 (red)) mutation rate. The net change in affinity is determined by calculating the difference between the number of beneficial mutations and the number of deleterious mutations. Among the 64 possible progeny cells, 25% of B cells exhibit equal or improved affinity in the constant mutation rate scenario, compared with 50% in the decreasing mutation rate scenario. **c**, Probability distribution of B cell node sizes in the GC for two scenarios: constant *p*_mut_ = 0.5 (black) and decreasing *p*_mut_ (red). Source Data

**Fig. 2:**
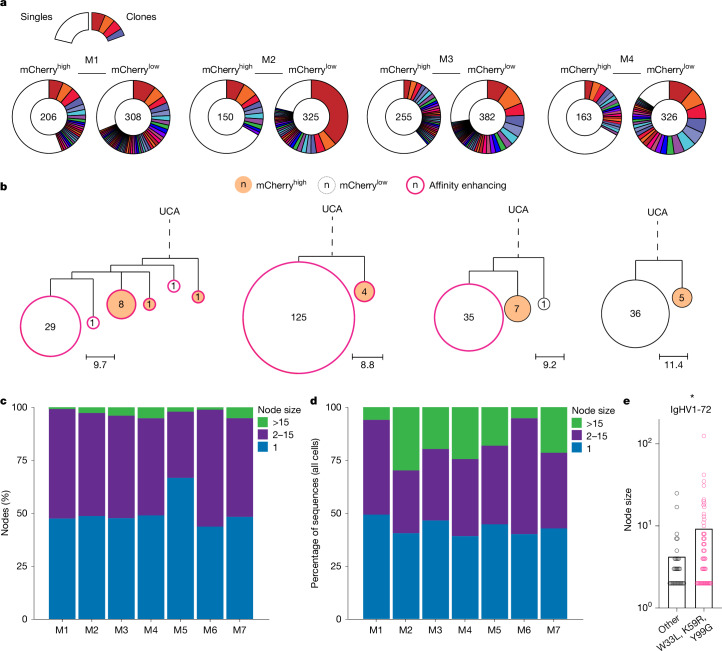
Affinity maturation in NP-OVA elicited GC reactions. **a**, Pie charts depicting clonal distribution of antibody sequences obtained from mCherry^high^ or mCherry^low^ NP-OVA elicited GC B cells from four of *n* = 7 mice analysed (M1–4). Numbers inside charts indicate the total sequences analysed per compartment. Coloured slice sizes are proportional to the number of clonally related sequences; white slices represent singles (sequences isolated only once). **b**, Representative genotype-collapsed phylogenetic trees containing nodes with more than 15 identical members. UCA (inferred) sequences appear at the root of the tree, connected (dashed line) to observed sequences (nodes) by branching. Sub-branching (solid line) reflects mutational distance between observed sequences (nodes). Scale bars represent mutational distance in nucleotides (per tree). Circle centres display the number of identical sequences in a node. Pink outlined nodes indicate sequences carrying any one of the affinity-enhancing mutations (W33L, K59R, Y99G). mCherry^high^ and mCherry^low^ nodes are filled red-orange and white, respectively. **c**, Bar graph showing the percentage of nodes containing 1 (blue), 2–15 (purple) or more than 15 (green) identical sequences among expanded clones for each of the 7 mice analysed. **d**, Bar graph showing percentage of total sequences that contributed to nodes containing 1 (blue), 2–15 (purple) or more than 15 (green) when analysis is extended to all cells (clones and singles). **e**, Graph displays size distributions of nodes with two or more identical sequences (derived from cells expressing IgHV1-72, paired with a lambda (λ) light chain) for cells with any (pink) or without (black) affinity-enhancing mutations (W33L, K59R, Y99G). *, Welch’s *t*-test (two-sided) shows significance (*P* < 0.05). Source Data

**Fig. 3:**
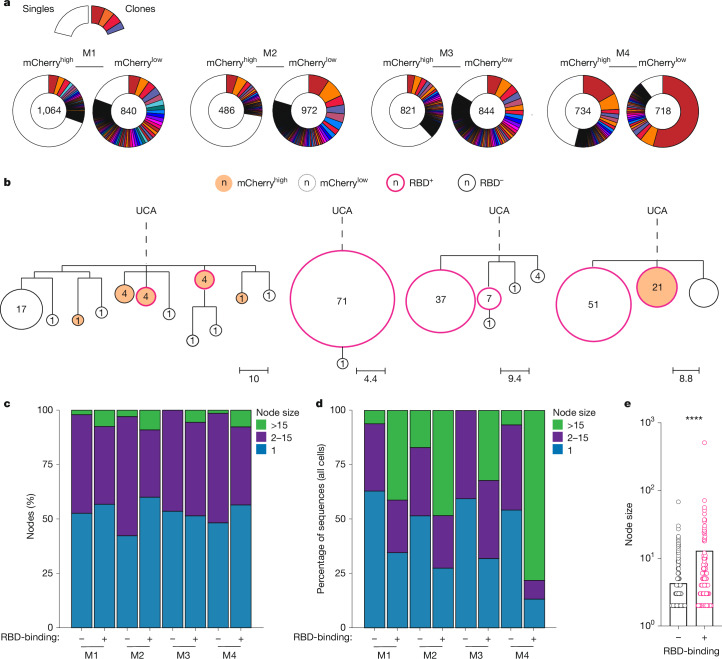
Affinity maturation in SARS-CoV-2 RBD-elicited GC reactions. **a**, Pie charts depicting clonal distribution of antibody sequences obtained from mCherry^high^ or mCherry^low^ RBD-elicited GC B cells from *n* = 4 of the mice analysed. Numbers inside charts indicate the total sequences analysed per compartment. Coloured slice sizes are proportional to the number of clonally related sequences; white slices represent singles (sequences isolated only once). **b**, Representative genotype-collapsed phylogenetic trees as in Fig. 2b. Sequences obtained from RBD^+^ and RBD^−^ sorted cells are outlined in pink and black, respectively. mCherry^high^ and mCherry^low^ nodes are filled red-orange and white, respectively. **c**, Bar graph showing the percentage of nodes containing 1 (blue), 2–15 (purple) or more than 15 (green) identical sequences in the RBD^+^ and RBD^−^ compartments among expanded clones for each of the four mice analysed. **d**, Bar graph showing percentage of total sequences that contributed to nodes containing 1 (blue), 2–15 (purple) or more than 15 (green) identical sequences among RBD^+^ and RBD^−^ fractions when all cells were considered (clones and singles). **e**, Graph showing size distribution of nodes containing two or more identical sequences among RBD^+^ (pink) and RBD^−^ (black) cells. ****P < 0.0001, by both unpaired Student’s *t*-test, (two-sided) and Welch’s *t*-test (two-sided, log-transformed). Source Data

**Fig. 4:**
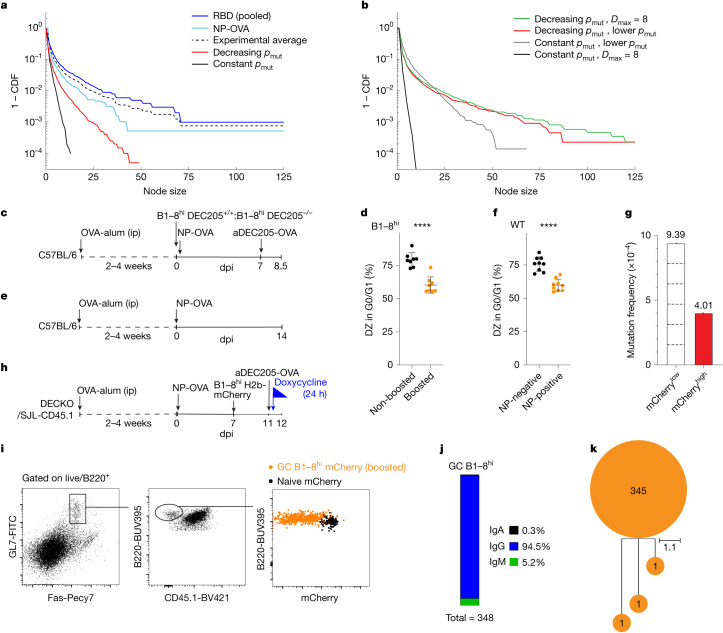
Reduced G0/G1 in high-affinity DZ B cells. **a**, Graph plotting CDF of node sizes from model simulations and experimental data. For the decreasing *p*_mut_ model (red), a linearly decreasing per division mutation probability as a function of number of divisions *D* from *p*_mut_(*D* = 1) = 0.6 to *p*_mut_(*D* = 6) = 0.2 was used, yielding ${\bar{p}}_{{\rm{mut}}}=0.48$, where ${\bar{p}}_{{\rm{mut}}}$ is the average per division mutation probability experienced. Averages over ten simulation runs are plotted. **b**, Graph comparing simulated node size CDF for alternate choices in the maximum number of divisions and mutation probability. Decreasing *p*_mut_ models where the maximum number of divisions is eight, *D*_max_ = 8, with linearly decreasing mutation probability from *p*_mut_(*D* = 1) = 0.6 to *p*_mut_(*D* = 8) = 0.12 yielding ${\bar{p}}_{{\rm{mut}}}=0.44$ (green), and decreasing *p*_mut_ model with *D*_max_ = 6 but with linearly decreasing mutation probability from *p*_mut_(*D* = 1) = 0.6 to *p*_mut_(*D* = 6) = 0.1 yielding ${\bar{p}}_{{\rm{mut}}}=0.45$ (red) are shown. Constant *p*_mut_ models with lower *p*_mut_ = 0.2 (grey) and *p*_mut_ = 0.5 but with *D*_max_ = 8 (black) are shown. Average over five simulation runs plotted. **c**, Schematic representation of the experiment used in **d**. ip, intraperitoneal injection. **d**, Graph showing percentages of non-boosted B1–8^hi^ DEC205^−/−^ (black) or boosted DZ B1–8^hi^ DEC205^+/+^ (orange) B cells in G0/G1 among *n* = 8 mice assayed. ****Unpaired two-tailed Student’s *t*-test comparing non-boosted and boosted shows significance (*P* > 0.0001). **e**, Schematic representation of the experiment shown in **f**. **f**, Graph showing percentage of NP non-binding (negative-black) or NP-binding (positive-orange) DZ B cells in G0/G1 among *n* = 9 mice assayed. ****Unpaired two-tailed Student’s *t*-test comparing NP-negative and NP-positive fractions shows significance (*P* > 0.0001). **g**, Re-analysis of SHM within JH4 intron of mCherry^low^ or mCherry^high^ GC B cells^23^. Dashed lines indicate divisions. Experiments were performed at least twice, error bars plot mean and s.d. **h**, Schematic for **i**–**k**. **i**, Representative flow cytometric plots profiling mCherry dilution from B1–8^hi^ H2b-mCherry GC (orange) or naive B cells (black) 24 h after boost; 348 GC B1–8^hi^ H2b-mCherry DEC-205^+/+^ VH regions sequences were retrieved across the six mice assayed. **j**, Bar chart interrogating isotype switching. **k**, Single tree visualizing SHM in V_H_ regions of the 348 sequences. Unmutated sequences appear at the root, and diversified progeny appear as sub-branching. dpi, days post immunization. Source Data

Our agent-based model makes the assumptions that competition among LZ B cells is mediated by affinity-dependent acquisition of antigens from follicular dendritic cells (FDCs) (Fig. 1a, (1)), and subsequent selection by T_FH_ cells contingent on antigen presentation (Fig. 1a, (2)). Selected LZ B cells then migrate to the DZ and undergo a programmed number of divisions (*D*), which is proportional to the magnitude of T_FH_ cell help received (Fig. 1a, (3)). Each division is accompanied by SHM with a probability of mutation (*p*_mut_ ≈ 0.5)^18^, where mutations can be silent (*p*_sil_ = 0.5), lethal (*p*_let_ = 0.3) (such as those that cause loss of B cell receptor (BCR) expression), affinity deleterious (*p*_del_ = 0.19) or affinity enhancing (*p*_enh_ = 0.01) on the basis of previously determined experimental and computational probabilities^19–21^. Following division, DZ B cells migrate back to the LZ for further rounds of affinity-based selection and then the cycle starts over (Fig. 1a, (4))^6,22,23^. A detailed description of the model and the parameters used in simulations can be found in Methods and in Supplementary Table 1.

Due to the relative paucity of beneficial mutations, generation of high-affinity B cells is a rate-limiting step in affinity maturation. Clonal dominance arises in GCs by strong expansion of high-affinity somatic variants from within a clonal family, generating diversified progeny and/or small collections of identical cells that collectively augment affinity maturation^16^. Despite being observed in various settings^16,24–27^, how identical somatic variants are generated or their overall contribution to affinity maturation has not been elucidated. To understand the behaviour of high-affinity B cells and their progeny, we simulated a clonal burst in the case where the mutation probability per division *p*_mut_ is a fixed value (constant *p*_mut_ = 0.5) regardless of the prescribed division (*D*) in the DZ (Fig. 1b). Specifically, we looked at the change in affinity among the progeny of GC B cells undergoing a maximum of six consecutive cell divisions in the DZ (Fig. 1b). In this constant −*p*_mut_ simulation, progeny of a DZ B cell dividing six times experienced generational degradation of affinity by the accumulation of deleterious mutations^19^. This, and the added probability of acquiring lethal mutations, predicted that, out of a possible 64 progeny, six divisions produced on average only 27 cells, and that more than 40% of these exhibited lower affinities than their parent. We concluded that clonal expansion at a constant rate of SHM would generate many unfit progeny and would not obviously augment affinity maturation.

To address the problem of ‘backsliding’ or affinity degradation, we explored a scenario where the probability of mutation per division, *p*_mut_, is dependent on the magnitude of T_FH_ cell help received in the LZ. Specifically, we considered the case where the mutation probability (*p*_mut_) for B cells signalled to divide (*D*) consecutive times in the DZ, *p*_mut_(*D*), decreases linearly from *p*_mut_(*D* = 1) = 0.6 to *p*_mut_(*D* = 6) = 0.2, which would correspond to a threefold decrease in mutations per division for the progeny of high-affinity B cells. Decreasing *p*_mut_ for DZ B cells dividing six times resulted in an increased average of 41 progeny cells, with now only 22% of these having lower affinity than their parent (Fig. 1b). Therefore, an affinity-dependent *p*_mut_ can theoretically facilitate the preferential establishment of high-affinity B cells in the GC, without extensive generational ‘backsliding’ in affinity.

To examine whether affinity-dependent rates of mutation facilitate establishment of expanded high-affinity B cell somatic variants, we employed our agent-based model to study the size of identical B cell clones within the GC. Throughout the simulations, we tracked the mutation history of each B cell, allowing us to identify genetically identical B cells, or nodes (Fig. 1c). We found that, with a constant *p*_mut_ (Fig. 1c, black), groups of identical B cells did not exceed 15 identical members. In contrast, decreasing *p*_mut_ with respect to T_FH_ cell help (Fig. 1c, red) produced remarkably larger populations of identical B cells, displaying a long-tailed distribution. Thus, a model in which T cell help modulates mutation rates favours the emergence of large groups of genetically identical high-affinity B cells (nodes).

**Extended Data Fig. 1:**
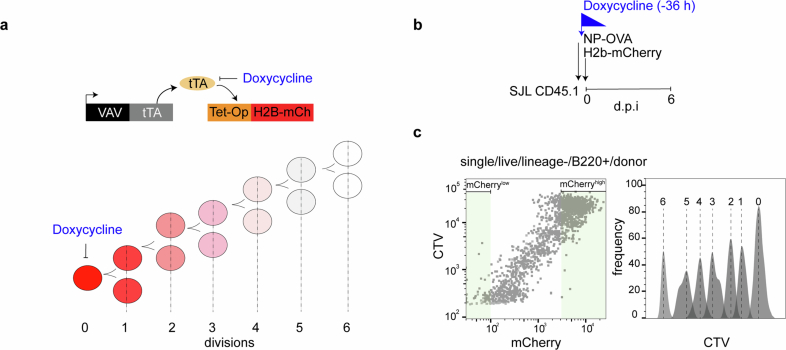
Tracking cell division using the H2b-mCherry system^23^. **a**, Diagrammatic representation of the Vav-tTA and Tet-Op-H2b-mCherry transgenes that were combined (tTA-H2b-mCherry mice) to trace up to 6 cell divisions after doxycycline exposure as described previously^22,40^. **b**, Schematic representation of the experiment shown in Extended Data Fig. 1c. Cell trace violet (CTV) labelled H2b-mCherry B cells were transferred into B6.SJL.CD45.1 hosts at the time of NP-OVA immunization and doxycycline exposure. CTV and H2b-mCherry dilution within congenic H2b-mCherry transferred cohorts was then assayed by flow cytometry 6 days later. **c**, Left, representative flow cytometry profiling mCherry fluorescence versus CTV dilution in dividing B cells. Filled boxes, highlight the number of cellular divisions (as measure by CTV) captured in mCherry^high^ (division ≤1) and mCherry^low^ (divisions≥6) fractions respectively. Right, Histogram profiling 6 cell division CTV peaks and one undivided, as indicated by the dashed lines. Source Data

To experimentally test whether mutation rates are variable, and dependent on both affinity and division rates, we tracked GC B cell division in mice that express mCherry labelled Histone-2b (H2b-mCherry) under the control of a doxycycline (DOX)-sensitive promoter^23,28^. Lymphocytes from these mice (H2b-mCherry mice) constitutively express the mCherry indicator. Administration of DOX turns off the reporter gene and, upon dividing, cells dilute the indicator in proportion to the number of divisions made, whereas quiescent cells retain the indicator^22,23,28^ (Extended Data Fig. 1a–c).

**Extended Data Fig. 2:**
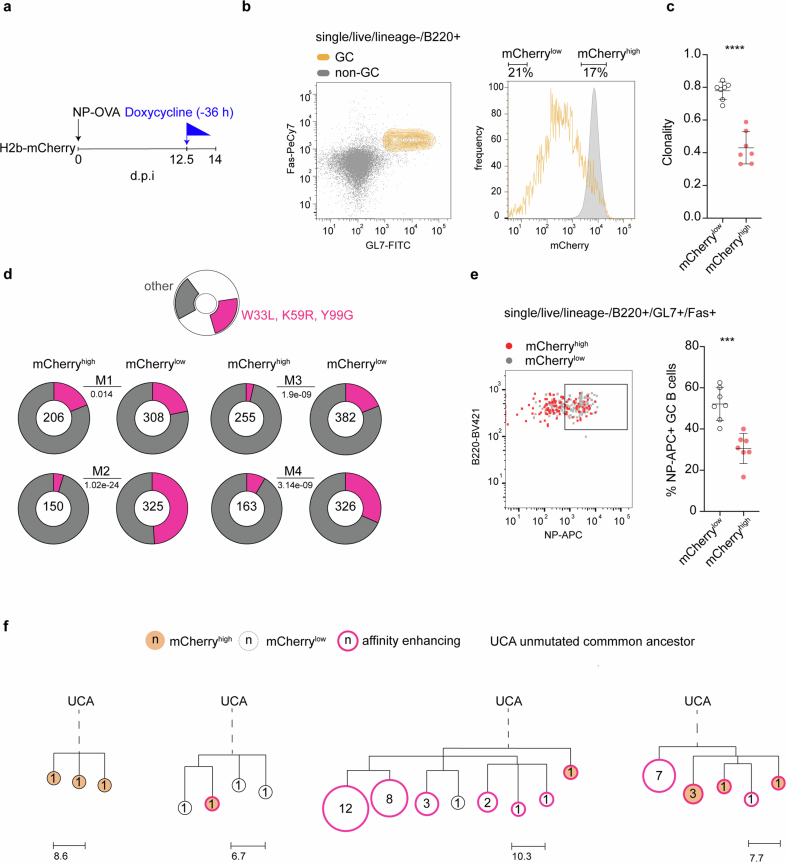
Analysis of NP-OVA elicited GCs. **a**, Schematic representation of the experiment shown in Fig. 2 and Extended Data Fig. 2b–f. Mice were immunized with NP-OVA and injected with doxycycline (DOX) 36 h before assay **b**, Left, Representative flow cytometry plots profiling GC B cells (orange). Right, histogram compares relative H2b-mCherry fluorescence among GC B cells (orange trace) as compared to naïve quiescent B cells (grey trace). **c**, Graph shows clonality observed among mCherry^low^ or mCherry^high^ cells. **** Unpaired Student’s *t*-test, (two-sided) shows significance. **d**, Pie charts depict the fraction of GC B cells that do (pink) or do not (grey) carry affinity enhancing mutations among the mCherry^low^ or mCherry^high^ GC B cells analyzed by sc-RNA sequencing. Numbers inside of the charts indicate the total sequences analyzed per compartment. Values outside of the pie charts are the respective p-values as calculated by Fisher’s exact test (two-sided). **e**, Right, Representative flow cytometry plots profiling NP-binding status of GC mCherry^high^ (pink) and GC mCherry^low^ (grey) isolated compartments from the same mouse. Left, Graph depicts the frequency of NP-bait binding in the mCherry^low^ or mCherry^high^ cells from the n = 7 mice analyzed in the sc-RNA sequencing in Fig. 2. ***Unpaired Student’s *t*-test, (two-sided) shows significance (p < 0.002). **f**, Representative genotype-collapsed phylogenetic trees containing nodes<15 as described in Fig. 2b. Source Data

To track GC B cell division in vivo, we immunized H2b-mCherry mice with 4-hydroxy-3-nitrophenylacetyl conjugated to ovalbumin (NP-OVA), administered DOX on day 12.5 and assayed mCherry indicator dilution (Extended Data Fig. 2a,b). At 36 h after DOX administration on day 14, 17% of GC B cells were mCherry^high^ and 21% mCherry^low^, respectively, representing GC B cells that had divided on average one or fewer times or at least six times (Extended Data Figs. 1c and 2b).

To characterize the relationship between SHM and cell division, we purified mCherry^high^ and mCherry^low^ GC B cells from the popliteal lymph nodes of NP-OVA-immunized DOX-treated mice and performed single-cell mRNA sequencing (scRNA-seq) using the 10X Chromium platform. To profile clonality, we used paired immunoglobulin heavy (IgH)- and light (IgL)-chain sequences that resolved families of related cells (clones) derived from common ancestors. Consistent with their higher levels of cell division, mCherry^low^ populations were significantly more clonal than the corresponding mCherry^high^ populations (Fig. 2a and Extended Data Fig. 2c). As surrogates for affinity, we compared the frequency of anti-NP affinity-enhancing mutations (W33L, K59R and Y99G in cells using IgHV1-72 (ref. ^29^)) and measured NP-fluorophore binding by flow cytometry. On average, GC B cells that had undergone more divisions (mCherry^low^) were significantly enriched for cells with affinity-enhancing mutations and measurable NP-fluorophore binding compared with those that divided less (mCherry^high^) (Extended Data Fig. 2d,e). Therefore, cells that divided the greatest number of times showed significantly higher affinity for antigen compared with cells that divided less.

Despite being derived from the same unmutated common ancestor (UCA), expanded clones are heterogeneous, composed of a collection of diversified somatic variants (nodes) that have accumulated additional point mutations due to SHM. Genotype-collapsed phylogenetic trees were produced to visualize the contribution of individual somatic variants to each of the expanded clones (Fig. 2b and Extended Data Fig. 2f)^21,30^. UCAs are shown at the roots of the trees and are connected to variants through branching, as indicated by dotted lines. Sub-branching (solid lines) illustrates mutational distance between variants. Division, as measured by mCherry dilution and affinity-enhancing mutations (pink outline), were also mapped onto the tree to annotate cell division status and relative affinity (Fig. 2b and Extended Data Fig. 2f).

Our modelling predicts that a constant mutation rate of around 1 × 10^−3^ per base pair per cell division^1^, equivalent to approximately 50% chance of mutation per division *p*_mut_ = 0.5, should produce branched trees containing limited small collections of identical sequences (nodes) of 15 or fewer cells (Fig. 1c, black). Alternatively, the decreasing *p*_mut_ model, with otherwise identical simulation parameters, predicted trees with nodes that extended up to 50 cells (Fig. 1c, red). Analysis of the experimental data revealed trees with nodes comprised of 2–15 members as well as trees with much larger nodes that contained 15–125 identical members (Fig. 2b and Extended Data Fig. 2f). These grossly expanded nodes (more than 15) could not be accounted for by the constant *p*_mut_ model but were more consistent with the decreasing *p*_mut_ model.

Among the expanded clones, the fraction of nodes containing either 1, 2–15 or more than 15 genetically identical sequences were 50%, 47% and 3%, respectively (Fig. 2c and Supplementary Table 2). Thus, only a small fraction of all nodes have more than 15 identical sequences. However, when all sequences were considered independently, on average 18% of all GC B cells were found in nodes that carry more than 15 identical sequences (Fig. 2d and Supplementary Table 3). Therefore, mutation-free clonal bursts produce a large assortment of identical progeny that contribute to the GC reaction.

To examine how affinity might impact node formation and size, we compared nodes from IgHV1-72 (heavy chain that is associated with optimal NP-binding activity^31^) GC B cells that had or had not yet acquired one of the affinity-enhancing mutations (W33L, K59R, Y99G). Larger nodes were enriched among GC B cells carrying affinity-enhancing mutations (Fig. 2e). In conclusion, the experimental data are in keeping with the theoretical model suggesting that the GC reaction is optimized by mutation-free proliferative bursts of cells expressing high-affinity antibodies.

**Extended Data Fig. 3:**
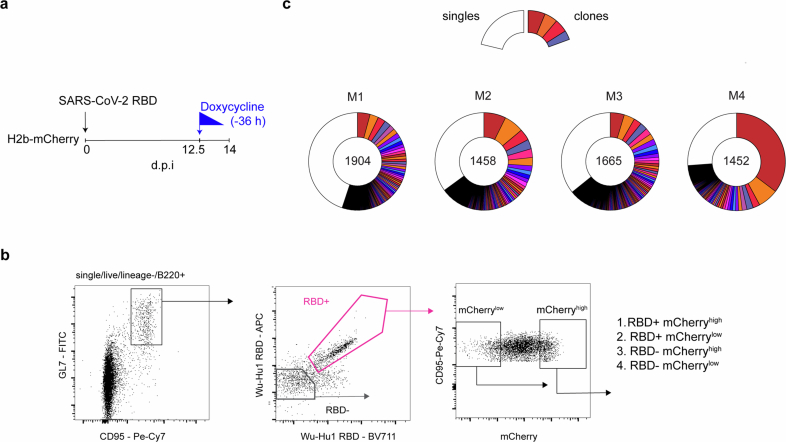
Profiling mCherry^high^ and mCherry^low^ fractions from SARS-CoV2 RBD induced GC B cells. **a**, Schematic representation of the experimental setup in Fig. 3. **b**, Representative flow cytometry plots showing the gating used to isolate on mCherry^high^ and mCherry^low^ compartments among RBD+ or RBD- GC B cells 14 days post immunization (d.p.i) with SARS-CoV-2 RBD (RBD) and 36 h after doxycycline exposure. GC cells were sorted without bias for RBD+ or RBD- **c**, Pie charts depict clonal distribution of antibody sequences in n = 4 mice analyzed. Numbers inside of the charts indicate the total sequences analyzed. Colored slice sizes are proportional to the number of clonally related sequences; white slices represent singles (sequences isolated only once). One mouse was excluded from analysis due to too few sequences being retrieved. Source Data

Immune responses to simple haptens like NP might differ from more complex protein antigens. To determine the contribution of mutation-free clonal bursts to immunization with a vaccine antigen, we performed single-cell analysis of GC B cells obtained from draining lymph nodes of H2b-mCherry mice immunized with the receptor binding domain (RBD) of SARS-CoV-2 in adjuvant (Extended Data Fig. 3a). GC B cells obtained 14 days after vaccination, and 36 h after DOX exposure were barcoded according to RBD-binding and mCherry expression, allowing paired analysis of sequence identity, division status and RBD-binding as a surrogate for affinity (Extended Data Fig. 3b). Genotype-collapsed phylogenetic trees were produced using paired IgH- and IgL-chain sequences from expanded clones (Fig. 3a,b and Extended Data Fig. 3c). RBD-binders (RBD^+^, pink outline), and RBD-non-binders (RBD^−^, black outline) were annotated along with cells’ mCherry expression (filled) (Fig. 3b). Similar to NP-OVA immunization, we observed large nodes containing identical sequences, indicating that extensive mutation-free clonal bursts occurred during SARS-CoV-2 vaccination (Fig. 3a–c). When only expanded clones were considered, the fraction of nodes with 1, 2–15 and more than 15 identical sequences represented 51%, 46% and 3% of all the nodes, respectively (Fig. 3c,d and Supplementary Table 4). Since large nodes contribute disproportionately, when all cells were considered independently, on average 24% of all GC B cells were derived from nodes that carry more than 15 identical sequences (Fig. 3d and Supplementary Table 5). Notably, the fraction of nodes containing more than 15 identical sequences was always greater among RBD^+^ as compared to RBD^−^ cells (Fig. 3d,e (*P* < 0.0001) and Supplementary Table 5).

**Extended Data Fig. 4:**
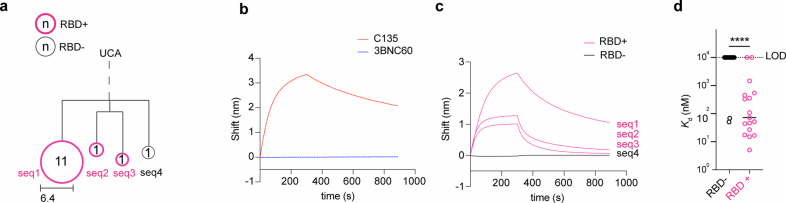
SARS-CoV-2 RBD antibody affinity measurements using bio-layer interferometry. **a**, One representative genotype-collapsed phylogenetic trees (of 8) that was selected, containing RBD+ and RBD- nodes, used to template for Fabs later used to measure antibody affinities using bio-layer interferometry (BLI) as in c-d. Example BLI traces for the binding and dissociation of **b**, control or **c**, experimental Fabs to biotinylated-RBD protein from tree depicted in a. Each curve represents one antibody. In b, the red dotted line denotes the binding of positive control C135 which is a potent neutralizing anti-RBD antibody. In b, blue dotted line denotes the trace of a negative control 3BNC60 Fab templated from an anti-HIV-1 antibody. In c, the pink solid lines denote the BLI traces from Fabs templated from RBD-binders featured in the genotype-collapsed phylogenetic tree in a. In c, the black solid line denotes the BLI trace from the RBD-non binders as in a. **d**, *K*_d_ values derived from Fabs templated from RBD- nonbinders (non-fill) and RBD+ binders (pink) as annotated from the genotype-collapsed phylogenetic trees and assigned by bait binding at the time of cell sorting. Dashed line annotated the limited of detection (LOD). **** Mann–Whitney *U*-tests (two sided) shows significance comparing RBD+ and RBD- cohorts (p < 0.0001). Source Data

To verify this enrichment and confirm that RBD-binding reliably reports on relative affinity, we produced fragment antigen-binding region (Fabs) templated from cells belonging to RBD^+^ and RBD^−^ nodes from expanded clones and measured affinity constants (*K*_d_) by bio-layer interferometry (Extended Data Fig. 4a–d). With only two exceptions, RBD^+^ nodes all produced high-affinity antibodies and RBD^−^ nodes showed little or no measurable affinity. When all *K*_d_ values were considered, antibodies from RBD^+^ nodes were significantly higher in affinity (lower *K*_d_s) than those from RBD^−^ nodes (Extended Data Fig. 4d; *P* < 0.0001). Thus, protein and hapten immunization are similar with respect to production of expanded nodes of high-affinity cells with identical sequences.

**Extended Data Fig. 5:**
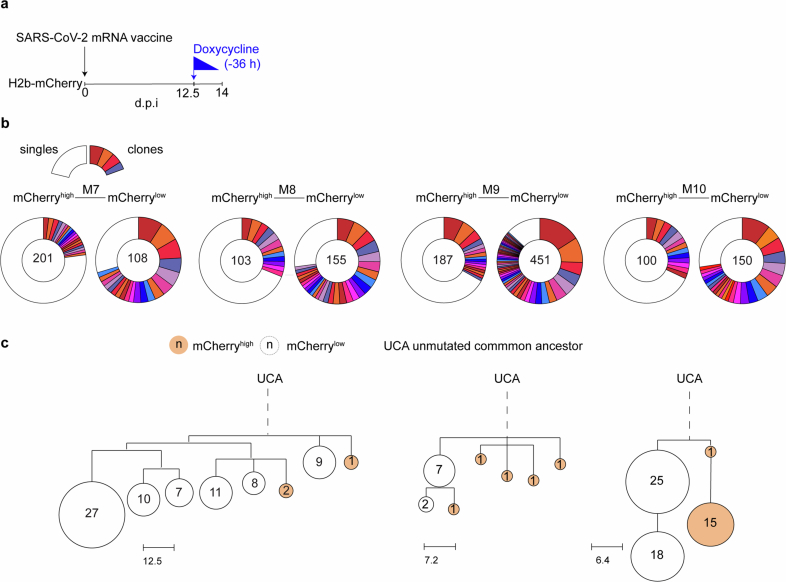
Expanded and grossly expanded node formation in GC fractions elicited by SARS-COVID-19 mRNA vaccination. **a**, Schematic representation of the experimental setup in Extended Data Fig. 4. Briefly, mCherry^high^ and mCherry^low^ compartments were isolated 14 days post immunization with SARS-CoV-2 mRNA vaccine and 36 h after doxycycline exposure **b**, Pie charts depict clonal distribution of antibody sequences in mCherry^high^ and mCherry^low^ cohorts from n = 4 vaccinated mice analyzed. Numbers inside of the charts indicate the total sequences analyzed per compartment. Colored slice sizes are proportional to the number of clonally related sequences; white slices represent singles (sequences isolated only once). **c**, Representative genotype-collapsed phylogenetic trees as in Figs. 2–3, highlighting tree featuring grossly expanded nodes (>15). Source Data

To determine whether these effects were adjuvant specific, we profiled GC B cells responding to SARS-CoV-2-mRNA vaccination (Extended Data Fig. 5a). GC B cells were obtained 14 days after mRNA vaccination, and 36 h after DOX exposure and isolated according to mCherry status. Single-cell sequencing was performed to resolve paired IgH- and IgL-chain sequences. Genotype-collapsed phylogenetic trees obtained from the expanded clones confirmed the contribution of large nodes to affinity maturation (Extended Data Fig. 5b,c). Thus, expanded cells with identical sequences arise in GCs elicited by different immunogens and adjuvants.

**Extended Data Fig. 6:**
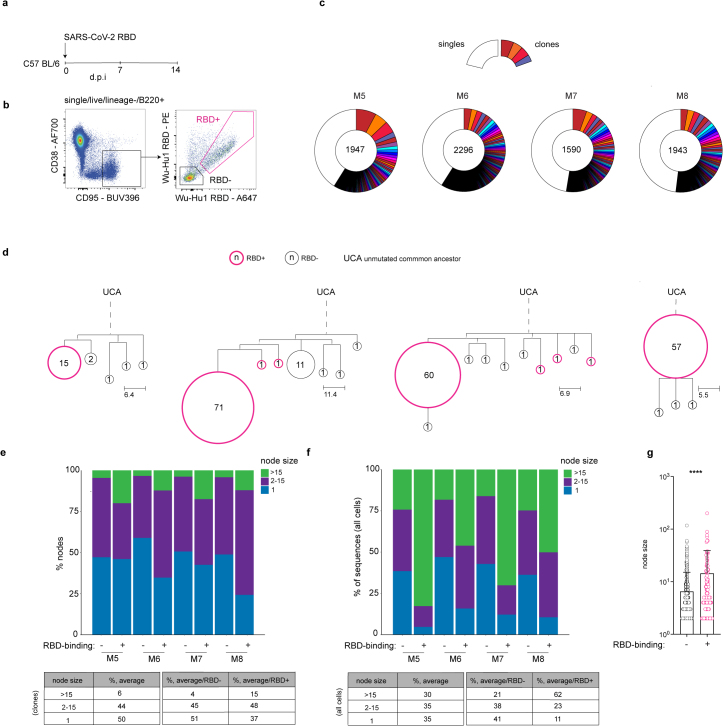
Affinity maturation in gross GC reactions elicited by SARS-CoV2 RBD immunization. **a**, Schematic representation of the experimental setup**. b**, Representative flow cytometry plots profiling RBD-binding among all GC B cells 14 days post immunization with SARS-CoV2 RBD. GC cells were sorted without bias for RBD+ or RBD- binders. **c**, Pie charts show clonal distribution of all antibody sequences obtained from each of the 4 mice analyzed. The number inside of the pie charts indicate the total number sequences retrieved per mouse. Colored slice size is proportional to the number of clonally related sequences and the white slices indicate the sum of the sequences isolated only once (singles). **d**, Representative genotype-collapsed phylogenetic trees as in Figs. 2–3. Sequences from nodes obtained from RBD+ and RBD- cells outlined in pink and black respectively. **e**, Bar graph shows the percentage of nodes containing 1 (blue), 2–15 (purple), or >15 (green) identical sequences in the RBD+ and RBD- compartments among expanded clones for each of the 4 mice analyzed. Table represents the averaged fraction of nodes containing 1 (blue), 2–15 (purple), or >15 (green) identical sequences in the RBD+ and RBD− compartments among expanded clones from the 4 mice. **f**, Bar graph shows percentage of sequences found in nodes containing 1 (blue), 2–15 (purple), or >15 (green) identical sequences among RBD+ and RBD− B cells when all cells were considered (i.e. clones and singles). Table represents the average fraction of sequences found in nodes containing 1 (blue), 2–15 (purple), or >15 (green) identical sequences between RBD+ and RBD− compartments, when all cells were considered. **g**, Graph shows size distribution of nodes containing 2 or more identical sequences among RBD+ (pink) and RBD− (black) cells. **** Welch’s *t*-test, (two-sided, log transformed) showed significance (p < 0.0001). Source Data

Profiling mCherry^high^ and mCherry^low^ B  cells is useful to compare cells that had divided one or fewer times versus at least six times following DOX exposure. However, as many GC cells were mCherry^intermediate^, many GC B cells were excluded from this analysis. To assess the contribution of mutation-free clonal bursts to total GC responses, we performed single-cell analysis of all GC B cells responding to RBD vaccination (Extended Data Fig. 6a–c). Consistent with the data obtained by mCherry fractionation, we observed large nodes containing identical sequences that contributed significantly to affinity maturation during SARS-CoV-2-RBD vaccination (Extended Data Fig. 6d–g). As expected, the grossly expanded nodes of B cells with more than 15 identical sequences also contributed disproportionately to the overall number of B cells and were highly enriched within high-affinity (RBD^+^) clones (Extended Data Fig. 6e–g). Thus, mutation-free clonal expansion contributes to affinity maturation in GC reactions across a range of immunological challenges.

To determine how the experimental data compared with the theoretical predictions we plotted the experimental cumulative distribution function (CDF) of node sizes alongside the simulations (Fig. 4a). Notably, the experimental results were closer to the node size distribution predicted by the decreasing *p*_mut_ model than the constant *p*_mut_ model. However, despite the qualitative similarities, the experimental cohorts displayed even longer tails not seen in the decreasing *p*_mut_ model. We reasoned that this discrepancy might be due to overly conservative assumptions about the maximum number of cell divisions per DZ cycle (six), which was inconsistent with the size of some of the experimentally observed nodes, or the choice of mutation probability for highly dividing B cells. Modestly changing the maximum number of cell divisions from six to eight produced a model that displayed a longer-tailed node size distribution and largely resolved the differences with respect to the experimental results (Fig. 4b, green). This change is more consistent with the experimental data that contained nodes with more than 64 cells in which more than six divisions have probably occurred. Alternatively, preserving the maximum number of divisions at six (*D* = 6), but decreasing the mutation rate (*p*_mut_) linearly from 0.6 to 0.1 in the decreasing −*p*_mut_ model also yielded a node size distribution consistent with experiments (Fig. 4b, red). In contrast, significantly lowering *p*_mut_ or, alternatively, extending the number of cell divisions in the constant *p*_mut_ model from six to eight did not yield a distribution of node sizes consistent with the experimental data (Fig. 4b, black and grey).

**Extended Data Fig. 7:**
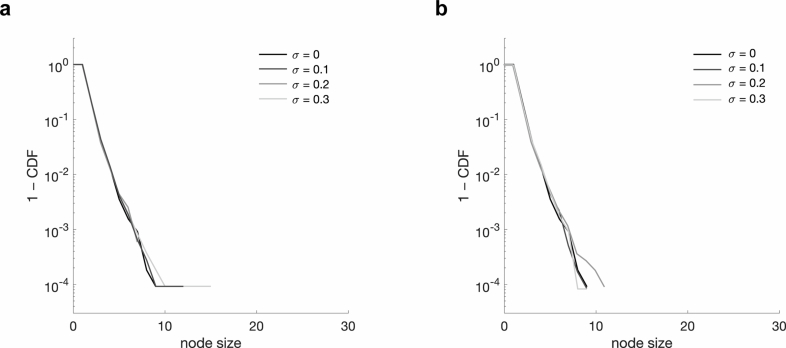
Node-size CDFs for constant *p*_mut_ simulations. **a**, Node-size CDFs for constant ${p}_{{\rm{mut}}}$ model with stochastic ${p}_{{\rm{mut}}}$. At each division event, the probability of mutation, ${p}_{{\rm{mut}}}$, was drawn from a normal distribution with mean $\mu =0.5$, and varying variance *σ*^2^. **b**, Node-size CDFs for constant ${p}_{{\rm{mut}}}$ model with stochastic T cell help. At each selection event, stochasticity in the number of divisions was induced by adding noise to the amount of acquired antigen by B cell (Eq. 1) as, ${A}_{{\rm{noisy}}}=A(1+\xi )$, where *A* is the amount of acquired antigen (see Methods), *ξ* is a random variable with a normal distribution with zero mean and varying variance *σ*^2^. For both a & b, the average over 5 simulation runs are shown. Source Data

Finally, we considered whether stochasticity in the constant *p*_mut_ model could account for the large nodes observed in experimental data. To this end, we simulated the case with stochastic *p*_mut_ (Extended Data Fig. 7a), and the case where the number of divisions corresponding to a constant level of T cell help was stochastic (Extended Data Fig. 7b). We found that neither scenario could account for the long-tail behaviour of node sizes observed in experiments. Thus, both the theory and experimental data are consistent with the idea that the per division rate of SHM is regulated and decreases with increasing T cell help.

**Extended Data Fig. 8:**
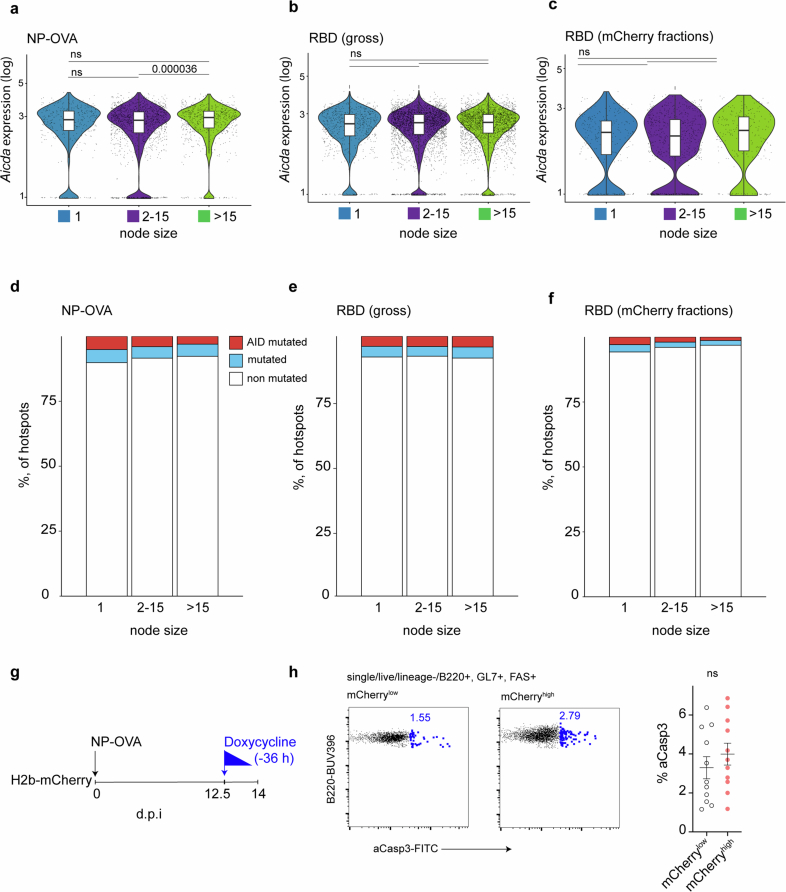
*Aicda* transcript expression and AID hotspot availability is similar between cells of different the node size classes. Violin plots plotting *Aicda* transcript levels by sc-RNA sequencing, of cells that contribute to nodes of size = 1 (blue), = 2–15 (purple), or >15 (green) in **a**, NP-OVA immunized H2b-mCherry mice (mCherry^high^ and mCherry^low^ pooled) or **b**, RBD immunized wildtype mice (gross) or **c**, RBD immunized H2b-mCherry mice (mCherry^high^ and mCherry^low^ fractions pooled). Welch’s *t*-test (two-sided) used to calculate significance or lack thereof. **d**, Bar graphs plot the relative frequency of AID genomic hotspots that are mutated (red and blue) or unmutated (white) in cells from nodes size = 1, = 2–15, or >15 in NP-OVA immunized H2b-mCherry mice (mCherry^high^ and mCherry^low^ pooled) or **e**, RBD immunized wildtype mice (gross) or **f**, RBD immunized H2b-mCherry mice (mCherry^high^ and mCherryl^ow^ fractions pooled). **g**, Schematic representation of the experimental setup in h. **h**, Left, representative flow cytometry plots profiling positive and negative (blue versus black dots respectively) aCaspase3 (aCasp3) staining within mCherry^high^ or mCherry^low^ GC B cells 14 days post immunization and 36 h after doxycycline exposure. Right, bar graphs plotting the frequency (y axis) of cells in the respective mCherry cohorts (x axis) that have measurable aCasp3 staining (blue dots). Each dot is an individual mouse, mean with s.e.m is plotted, experiments was performed three times (ns). Source Data

SHM is mediated by the enzyme activation-induced cytidine deaminase (AID)^2,14,32–35^. AID has relatively poor catalytic activity and even small changes to AID expression result in changes in the rate of SHM^36^. To determine whether variable mutation rates in GC B cells were mediated by altered AID expression in expanded nodes, we profiled its expression in NP-OVA and RBD-elicited GC B cells contributing to nodes of sizes 1, 2–15 or more than 15 (Extended Data Fig. 8a–c). Despite the absence of SHM in grossly expanded nodes, there were no significant decrease in *Aicda* transcript levels in the larger node classes. Thus, differential *Aicda* expression levels are not likely to be responsible for the absence of mutation in large nodes.

AID introduces C>U mutations at preferential nucleotide sequence hotspots (WRC, W = A/T, R = A/G, underline indicates residue targeted for mutation)^37,38^. To determine whether absence of SHM in expanded nodes was due to previous loss of these motifs, we profiled AID hotspots between cells contributing to nodes of sizes 1, 2–15 or more than 15. Hotspot motifs were equally intact in cells belonging to all three classes of nodes (average 92%, 93% and 94%, respectively; Extended Data Fig. 8d–f). Thus, target motif decay does not account for differences in SHM between nodes. Finally, we compared the level of cell death among mCherry compartments and found no significant differences (Extended Data Fig. 8g,h).

**Extended Data Fig. 9:**
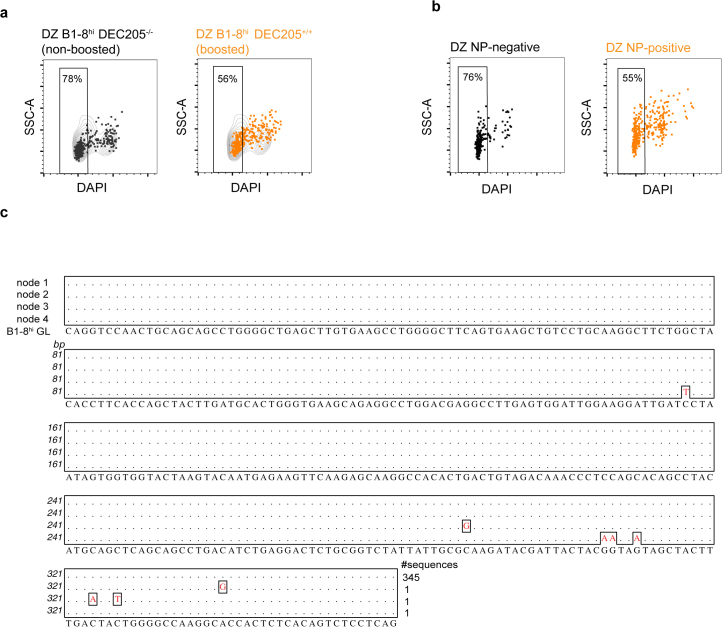
Reduced G0/G1, and SHM in high affinity GC B cells. **a**, Representative flow cytometry plots profiling DAPI staining, and G0/G1 occupancy among DZ B1–8^hi^ DEC-205^−/−^ (black) and DZ B1–8^hi^ DEC-205^+/+^ (orange) GC B cells 36 hr after exposure to anti-DEC205-OVA as in Fig. 4c,d. **b**, Representative flow cytometry plots profiling DAPI staining and G0/G1 occupancy among DZ NP non-binding (negative-black) and NP-binding (positive-orange) and GC B cells as in Fig. 4d. **c**, Alignment of the sequences observed in the four nodes (totally 348 cells) versus the unmutated IgHV B1–8^hi^ knock in reference sequences as referred to in Fig. 4h–k. Dots in sequence alignments are used to indicate matching nucleotides between sequences. Nucleotide mismatches from B1–8^hi^ germline (GL) reference sequence (bottom), produced by somatic mutations are annotated in red at the relevant base pair (bp). Source Data

SHM is cell cycle dependent and limited to the G0/G1 phases of the cell cycle, when AID is in spatial contact with genomic DNA^10–12,22,23^. High-affinity B cells receiving strong T cell help transit through the S phase of the cell cycle faster than their low-affinity counterparts, but the consequences on the G0/G1 phase of the cycle and on SHM have not been examined^22^. To determine how regulation of the cell cycle might contribute to variable, per division mutation rates, we initially used anti-DEC205 chimeric antibodies to deliver strong selection signals to GC B cells^23,39^. Congenic DEC205^+/+^ B1–8^hi^ B cells were adoptively transferred into OVA primed mice that were subsequently immunized with NP-OVA and later boosted with anti-DEC205-OVA chimeric antibodies or left non-boosted (Fig. 4c)^39,40^. Cell cycle analysis by DNA content revealed that B1–8^hi^ B cells that received strong selection signals (boosted, orange) and experienced a greater number of divisions were significantly less likely to be in G0/G1 than controls (non-boosted, black) (Fig. 4d and Extended Data Fig. 9a).

**Extended Data Fig. 10:**
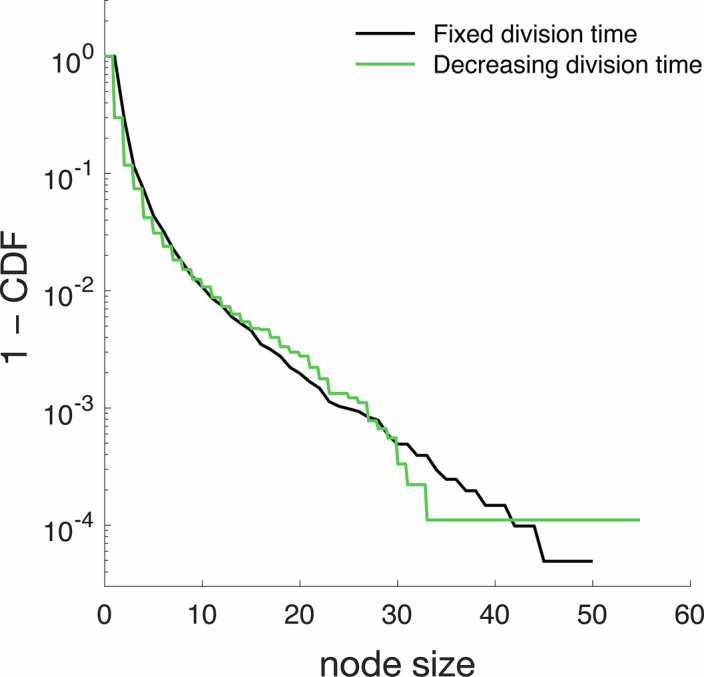
Node-size CDFs for simulations with varying B cell division time. Node-size CDFs for decreasing ${p}_{{\rm{mut}}}$ model (${p}_{{\rm{mut}}}(D=1)=0.6$ to ${p}_{{\rm{mut}}}({D}_{\max }=6)=0.2$) with constant B cell division time $({\tau }_{{\rm{div}}}=5{\rm{h}})$ in the DZ (black) and B cell division time that decreases proportionally with prescribed number of divisions, *D* (green) are shown. In the second scenario, B cell division time *τ*_div_ was chosen to decrease linearly from ${\tau }_{{\rm{div}}}(D=1)=12{\rm{h}}$ to ${\tau }_{{\rm{div}}}({D}_{\max }=6)=4{\rm{h}}$. The average over 5 simulation runs are shown. Source Data

To determine whether shorter time spent in G0/G1 was a feature of high- versus low-affinity B cells participating in polyclonal immune responses, we immunized OVA primed mice with NP-OVA and measured the cell cycle distribution of DZ cells that bound to NP-fluorescent bait by flow cytometry as a surrogate for affinity (Fig. 4e,f and Extended Data Fig. 9b; *P* < 0.001). The proportion of DZ NP-fluorophore binding cells in G0/G1 was significantly lower than non-binder DZ counterparts (Fig. 4f, *P* < 0.001). These results imply that high-affinity B cells cycle through the G0/G1 phase significantly faster than low-affinity B cells, shortening the time for AID mutator activity and thereby decreasing mutation probability per division, thus leading to the long-tailed behaviour of node sizes predicted by the decreasing *p*_mut_ model and as observed in our experiments. Notably, altering the speed of the cell cycle without changing the per division mutation probability in our simulations does not result in changes to the node size distribution (Extended Data Fig. 10).

To confirm that GC B cells that divide more also mutate less per cell division we re-examined mutation rates in the IgH *J*_*H*_*4* intron in DZ B cells from H2b-mCherry mice immunized with NP-OVA (Fig. 4g)^23^. The IgH *J*_*H*_*4* intron was selected because mutations in this region are not subject to selection. We observed that mCherry^low^ cells that had divided at least six times were only approximately 2- to 2.5-fold more mutated (not the expected sixfold) than their mCherry^high^ counterparts that had divided once or less, representing a roughly two- to threefold lower mutation rate per division in cells that undergo several rounds of division in the DZ (Fig. 4g)^23^.

To determine how the altered cell cycle might impact SHM, we delivered strong selection signals to GC B cells using anti-DEC205-OVA (ref. ^22^). Congenic DEC205^+/+^ H2b-mCherry B1–8^hi^ B cells that express high-affinity receptors for NP were adoptively transferred into polyclonal host mice that had been immunized with NP-OVA 7 days previously (Fig. 4h). Approximately 3.5–4 days after the transfer, enough time for B1–8^hi^ B cells to become activated and enter the GC but not enough for the accumulation of SHM^41^, we administered DOX and anti-DEC205-OVA to track cell division and deliver a strong selection signal, respectively (Fig. 4i)^23,39^. Although asynchronous, most DEC205^+/+^ B1–8^hi^ B cells underwent at least two divisions within the 24-h observation period, as compared with naive H2b-mCherry counterparts, as captured by respective mCherry dilution or retention (Fig. 4i). Despite division and class switch recombination, which would have occurred after activation and before GC entry^42^, B1–8^hi^ IgVH sequence analysis revealed only eight base pairs were mutated in a total 125,628 bases sequenced, generating only three mutated cells out of total of 348 cells (Fig. 4j,k and Extended Data Fig. 9c). Thus, the observed rate is significantly less than the accepted rate of one mutation per 10^3^ bp per division.

Together these data are consistent with the idea that strong selection signals decrease the relative time DZ cells spend in G0/G1, thereby reducing their exposure to AID and lowering their per division mutation rates.

## Discussion

SHM is an unusual feature of the antibody system. It is essential for affinity maturation but comes at the high cost of producing off-target DNA damage that can lead to chromosome translocations that are associated with malignancy^43,44^. Because mutation is random, it is also more likely to decrease antibody affinity than enhance it. Based entirely on theoretical considerations, we predicted that optimizing affinity maturation requires varying mutation rates such that high-affinity antibody-producing B cells that divide a greater number of times should mutate at a lower rate per division. The experimental data presented here support this theory.

SHM is mediated by AID^2,32,33^. The activity of this enzyme is regulated in a number of different ways including transcription, phosphorylation^13,45,46^ and nuclear access, the last of which is linked to cell division and specifically to the early G1 phase of the cell cycle after which AID is exported from the nucleus^10,11,45,47–49^. High-affinity LZ GC B cells that receive strong positive selection signals divide more rapidly^6^ than their lower affinity counterparts^22,23^. Although other mechanisms may also contribute to decreased SHM, the experimental data suggest that rapidly dividing cells limit mutation in part by spending less time in G0/G1, thereby reducing access of AID to the DNA. In addition to preferentially preserving B cells that express high-affinity antibodies, this mechanism also decreases the likelihood that rapidly dividing GC B cells suffer off-target AID-mediated DNA damage.

Our finding that high-affinity interactions lead to lower mutation rates may contribute to the observation that vaccines or vaccine boosts that select high-affinity antibody-producing cells favour their conservation during clonal expansion and thereby contribute to imprinting or original antigenic sin. Notably, the data suggest that vaccine strategies that aim to diversify immune responses to increase breadth or shepherd antibody responses by sequential vaccination to elicit highly mutated antibodies could benefit from boosting with lower affinity antigens.

## Methods

### Agent-based modelling of the GC reaction

In the agent-based model, we simplify GC B cells into four distinct states: B cells competing for antigen and T_FH_ signal in the LZ (Fig. 1a, (1)), B cells migrating from LZ to DZ (Fig. 1a, (2)), B cells undergoing proliferation and SHM in the DZ (Fig. 1a, (3)) and B cells migrating from DZ to LZ (Fig. 1a, (4)). To focus on the influence of a help-signal-dependent per division mutation rate on B cell affinity maturation, differentiation of B cells into effector cells and the export of effector cells from the GC are neglected.

Each GC B cell is represented by a vector that specifies its current state and essential parameters, such as BCR affinity for antigen, amount of acquired antigen and mutation history. We update the state of each B cell at every time step, governed by state-specific dynamics. Each LZ B cell (state 1) interacts with an FDC at a maximum rate of *r*_FDC_ and, upon interaction with an FDC, successfully acquires an antigen with probability *p*_antigen_ = (1 + *e*^⟨*a*⟩−*a*^)^−1^, where *a* is the affinity of its BCR for the antigen, and ⟨*a*⟩ is the average BCR affinity of all the B cells in the LZ at the current time step (state 1). If a B cell successfully acquires an antigen, its amount of acquired antigen increases by one unit. Simultaneously, each T_FH_ cell interacts with a random LZ B cell at a rate of *r*_TFH_. Upon each interaction event, the T_FH_ cell provides a selection signal to the B cell, where the magnitude of the selection signal *D* is dependent on the amount of acquired antigen, according to

1 $$D=\lfloor ({D}_{\max }+1)[1-{e}^{-(A-{A}_{\min })/{A}_{0}}]\rfloor ,$$

where *D*_max_ is the maximum number of consecutive divisions allowed in the DZ, *A* is the amount of acquired antigen, *A*_min_ and *A*_0_ set the stringency of T_FH_ cell selection and $\lfloor \cdot \rfloor$ denotes the floor function. If *D* ≥ 1, *D* is saved to the vector of the B cell, and the B cell switches to state 2. If a B cell does not acquire sufficient help signal from a T_FH_ cell within its lifetime *τ*_B_, then it undergoes apoptosis and is removed from the simulation.

Selected B cells in state 2 take a time *τ*_LZ→DZ_ to differentiate from an LZ phenotype B cell to a DZ phenotype B cell before migrating to the DZ, entering state 3. DZ B cells (state 3) divide *D* consecutive times, where each division takes *τ*_div_ to complete. Upon a division event, each daughter cell acquires a mutation with probability *p*_mut_(*D*), which may depend on the magnitude of the selection signal acquired in the LZ. Mutations can be lethal, silent (no effect on affinity), deleterious (decreases affinity by Δ*a* = 1), or advantageous (increases affinity by Δ*a* = 1). The mutation history of each B cell is tracked under the assumption that each mutation is unique (infinite allele limit). After completing *D* divisions, all surviving progeny switch to state 4, taking a time *τ*_DZ→LZ_ to differentiate back into LZ phenotype B cells, then return to the LZ (state 1).

### Simulation details

Each simulation begins with 250 LZ B cells in state 1 and 200 T_FH_ cells, and runs for a period of 20 days using a time step of 5 × 10^−3^ h. Note that the number of T_FH_ cells remains fixed throughout the simulation.

For constant mutation rate simulations, we use a mutation probability of *p*_mut_(*D*) = 0.5 for all *D*18,48,49, whereas in the decreasing mutation rate simulations, the mutation probability is chosen to be linearly decreasing, for example, between *p*_mut_(1) = 0.6 and *p*_mut_(*D*_max_ = 6) = 0.2, that is,

2 $${p}_{{\rm{mut}}}(D)=0.6-\frac{0.6-0.2}{{D}_{\max }-1}(D-1).$$

Upon a mutation event, lethal, silent, deleterious and enhancing mutations occur with probabilities *p*_let_ = 0.3, *p*_sil_ = 0.5, *p*_del_ = 0.19 and *p*_enh_ = 0.01, respectively^19^. Simulation parameters are listed in Supplementary Table 1.

### Parameter estimation

For the initially studied decreasing *p*_mut_ model, mutation probabilities *p*_mut_(1) = 0.6 and *p*_mut_(6) = 0.2 were inferred from experimental measurements by Gitlin et al.^23^, who found that B cells that underwent approximately six divisions in the DZ accumulated only twice the number of mutations compared with B cells that underwent a single division in the DZ. This implies that B cells undergoing six divisions have a per division mutation probability that is lower by a factor of three compared with B cells that undergo a single division.

The rate at which each T_FH_ cell interacts with a B cell, *r*_TFH_, was inferred from the rate at which each B cell interacts with a T_FH_ cell, *r*_B_, using the relation

3 $${r}_{{\rm{TFH}}}=\left(\frac{1}{f}-1\right){r}_{{\rm{B}}},$$

where *f* is the fraction of cells in the GC that are T_FH_ cells. Equation (3) was derived by assuming that the interaction between any two cells in the GC occurs at a rate *r*_C_, which is related to *r*_TFH_ and *r*_B_, respectively, as *r*_TFH_ = (1 − *f*)*r*_C_ and *r*_B_ = *fr*_C_. Combining the two expression yields equation (3). A T_FH_ cell fraction of *f* *=* 0.15 was assumed to yield *r*_TFH_ = 0.4 h^−1^ (Supplementary Table 1).

### Mice

Mice were housed at a temperature of 72 °F and humidity of 30–70% in a 12-h light/dark cycle with ad libitum access to food and water. Male and female mice aged 8–10 weeks at the start of the experiment were used throughout. C57BL/6J mice were purchased from Jackson Laboratories. H2b-mCherry mice, B1–8^hi^ and B1–8^hi^ *DEC205*^*−/−*^ mice were generated and maintained at Rockefeller University. All mouse experiments were performed under Institutional Review Board approved protocols. Sample sizes were not calculated a priori. Animals were age (6–10 weeks) and sex matched within experiments (both sexes were used between experiments). Given the nature of the comparisons, mice were not randomized into each experimental group and investigators were not blinded to group allocation.

### Immunizations and treatments

For NP-OVA immunization experiments, C57BL/6J or H2b-mCherry recipient mice (6–12 weeks old) were immunized with 50 μg of OVA intraperitoneally and boosted 2–4 weeks later via footpad injection with 20 μg NP17-OVA (Biosearch Technologies) both precipitated in alum. For COVID-19 vaccination experiments, C57BL/6J or H2b-mCherry recipient mice were immunized in the footpads with 20 μg monomeric RBD precipitated in alum or received 1 μg of COVID-19 BioNTech (Pfizer) mRNA vaccine intramuscularly and the associated popliteal or inguinal lymph (respectively) were harvested 14 days thereafter.

For H2b-mCherry dilution experiments, mice were administered DOX (doxycycline hyclate, Sigma) by intraperitoneal injection of 2 mg DOX in PBS. Draining lymph nodes were collected for flow cytometric analysis 36 h later. H2b-mCherry dilution was monitored by flow cytometry.

αDEC205-OVA chimeric antibodies were expressed transiently in Expi293F cells using the ExpiFectamine 293 Transfection Kit (Thermo Fisher Scientific). The supernatant was collected 7 days later and the chimeric antibodies were concentrated by ammonium sulfate precipitation. After centrifugation, the pellet was resuspended in PBS and affinity purified on Protein G columns (Protein G Sepharose 4 Fast Flow, catalogue no. 17-0618-05, GE Healthcare); 2 μg of chimeric antibody in PBS was injected into footpads of the recipient mice at indicated time points.

### B cell transfer

Single-cell suspensions were prepared from the spleens and lymph nodes of donor mice. Resting B cell suspensions were enriched using negative selection using Magnisort B cell enrichment kit (Thermo Fisher). Approximately 5 × 10^6^ B1–8^hi^ DEC205^+/+^ B cells: B1–8^hi^ DEC205^−/−^ (5 × 10^5^ Igλ^+^, NP-specific B cells) composed of the indicated populations were injected into recipient mice by intravenous injection.

### Flow cytometry

Single-cell suspensions were stained with antibodies directly conjugated to surface markers. Intracellular stains for DNA content analysis used 2% paraformaldehyde and commercially available permeabilization buffer, coupled to incubation with 4′,6-diamidino-2-phenylindoleantibodies. For RBD-staining, RBD-biotin (in-house) was incubated with Streptavidin (SA)-BV711, SA-PE, SA-BV421 and SA-A647 or 30 min, covered, before addition to suspension. Multi-colour cytometry was performed on the Symphony flow cytometer (BD Biosciences) and analysed with FlowJo v.10.4.2. All cells were sorted on the BD FACSymphony S6 system at greater than 95% purity.

### Cell sorting and barcoding

For the mCherry scRNA-seq experiments, individual mice were barcoded separately using commercially available TotalSeq anti-mouse Hashtag reagents. mCherry^high^ and mCherry^low^ fractions were subsequently sorted into separate tubes and indexed and run as separate lanes on the sequencer. All cells were sorted on the BD FACSymphony S6 system and sequenced without enrichment bias.

For scRNA-seq of RBD challenged mice, individual mice were separately barcoded using commercially available TotalSeq anti-mouse Hashtag reagents. RBD-binders and non-binders, were sorted into separate tubes, indexed and run as separate lanes on the sequencer. All cells were sorted on the BD FACSymphony S6 system and sequenced without enrichment bias.

For the B1–8^hi^ scRNA-seq experiment the 10X was run once, but two cohorts of recipients that were treated and harvested separately, totalling six independent mice (CD45.1 or DECKO) used. Sequences analysed were pooled between all six mice and analysed collectively, filtering for sequences derived from the IgHV1-72 knock-in B1–8^hi^ sequence.

### Preparation of immunogen bait

4-Hydroxy-3-nitrophenylacetic acid succinimide ester (NP-Osu, Biosearch Technologies) was conjugated to Alexa Fluor 647 Streptavidin (SA-A647) hapten:streptavidin molar ratio of 10:1 or 20:1. Hapten–protein conjugation ratios were calculated by measuring the absorbance value at 430 nm. Purified and Avi-tagged SARS-CoV-2 RBD was biotinylated using the Biotin-Protein Ligase-BIRA kit according to manufacturer’s instructions (Avidity). Biotinylated RBD was conjugated to SA-fluorophores (tetramerized) for use in flow cytometry.

### Fab production

Heavy and light chain eBlocks (IDT) were cloned into human Fab and kappa/lambda expression vectors by restriction cloning^50,51^. His_6_-tagged Fabs and kappa/lambda light chains were expressed by transient transfection in Expi293F cells (Thermo Fisher Scientific) and purified using Ni Sepharose 6 Fast Flow resin (Cytiva).

### Bio-layer interferometry

Bio-layer interferometry assays were performed as previously described^10^ using the ForteBio Octet Red instrument (ForteBio Data Acquisition software v.11.1.3.25) at 30 °C with shaking at 1,000 rpm. Kinetic analysis using Octet SAX Biosensors (Satorius 18-5117) with biotinylated wild-type RBD and human Fabs was performed as follows: (1) baseline, immersion for 60 s in buffer (1× Octet Kinetic buffer, Sartorius, 18-1105); (2) loading, immersion for 200 s in a solution with 200 nM biotinylated wild-type RBD; (3) baseline, immersion for 200 s in buffer; (4) association, immersion for 300 s in solution with Fabs and (5) dissociation, immersion for 600 s in buffer. Curve fitting was performed using a fast 1:1 binding model and the data analysis software from ForteBio (ForteBio Data Analysis HT v.11.1.3.50). Mean *K*_d_ were determined by averaging all binding curves that matched the theoretical fit with an *R*^2^ value of at least 0.75.

### RNA sequencing

For scRNA-seq of NP-OVA challenged mice, single-cell suspensions were prepared from popliteal lymph nodes on day 14 after immunization. Samples were indexed with TotalSeqC (BioLegend) cell surface antibodies and live, lineage^−^, B220^+^, GL7^+^, Fas^+^, mCherry^high^ and mCherry^low^ GC cells were purified by flow cytometry and loaded onto a Chromium Controller (10x Genomics). scRNA-seq libraries were prepared using the Chromium Single Cell 5′ v.2 Reagent Kit (10x Genomics) according to the manufacturer’s protocol. Libraries were loaded onto an Illumina NextSeq with the mid-Output Kit (150 paired end) for V-D-J analysis or NOVAseq for single-cell gene expression. Hashtag indexing was used to demultiplex the sequencing data and generate gene–barcode matrices, respectively.

For scRNA-seq of RBD challenged mice, single-cell suspensions were prepared from draining lymph nodes on day 14 after immunization. Samples were indexed with TotalSeqC (BioLegend) cell surface antibodies and live, lineage^−^, B220^+^, CD38^−^, Fas^+^, RBD^−^ and RBD^+^ GC cells were purified by flow cytometry and loaded onto a Chromium Controller (10x Genomics). On average, 696 sequences were analysed per mouse.

### Single-cell library processing

scRNA-seq and Hashtag-oligos unique molecular identifier quantification were performed with Cell Ranger multi v.7.1.0 (10x Genomics), using the Cell Ranger GEX reference mm10, and analysed in R with Seurat v.4.3.0 (ref. ^52^). Cells were demultiplexed with MULTISeqDemux, and those classified as doublets or with mitochondrial content greater than 10% and feature count less than 200 or greater than 2,500 were excluded. Sample batches were then merged, scaled and normalized with SCTransform. Single-cell BCR libraries were mapped to the Cell Ranger VDJ GRCm38 reference using Cell Ranger multi v.7.1.0. The contigs containing less than 50 reads and more than one heavy or light chain were removed. For B1–8^hi^ scRNA-seq analysis, only IgHV1-72 heavy chains that derived from the B1–8^hi^ germline knock-in sequence were filtered for further analysis.

### Computational analyses of antibody sequences

Single-cell heavy and light chains were paired and analysed using igpipeline v.2.0 (https://github.com/stratust/igpipeline/tree/igpipeline2_timepoint_v2), as previously described^53^, using the mouse IMGT database as a reference^54^. The paired IgH and IgL chains of antibodies from the same clonal progeny were merged and aligned to the mouse IMGT germline sequence using mafft v.7.520 with default parameters^55^, except for --globalpair. Genotype-collapsed phylogenetic trees of clonal lineages were inferred using GCTree v.4.1.2 (https://github.com/matsengrp/gctree)^30^. Each node represents a unique IgH and IgL combination with number within each node indicating the number of identical sequences. The scales represent the branch lengths, estimated on the basis of the number of nucleotide mutations. AID hotspots regions, characterized by the motif WGCW (W = A/T)^56^, were mapped systematically across the germline sequences of each clone allowing for overlaps between hotspots. In the antibody sequences, these hotspots were classified as AID-mutated if G>A/C>T mutations were present, unmutated or containing other types of mutations.

### Classifications

Clones are defined as cells (clonal progeny) derived from a UCA (naive B cell). Expanded clones are composed of a heterogeneous collection of somatic variants, diversified from a common unmutated ancestor. Nodes within clones are cells with genetically identical IgH and IgL combinations.

### Statistical analyses

Statistical tests were conducted using R v.4.2.3 and/or Prism (GraphPad) software. Unpaired, two-tailed Student’s *t*-tests, two-tailed Fisher’s exact test and one-way analysis of variance with Tukey’s post hoc tests to further examine pairwise differences were used. Data were considered statistically significant at *P* ≤ 0.05, *P* ≤ 0.01, *P* ≤ 0.001 and *P* ≤ 0.0001. The number of mice per group, number of replicates and the nature of error bars are indicated in the legend of each figure. Centre bars always indicate mean and error bars always plot s.d. or s.e.m.

### Ethics statement

All procedures in mice were performed in accordance with protocols approved by the Rockefeller University Institutional Animal Care and Use Committee. All animal experiments were performed according to the protocols approved by the Institutional Animal Care and Use Committee of NIAID, NIH.

### Reporting summary

Further information on research design is available in the Nature Portfolio Reporting Summary linked to this article.

## Online content

Any methods, additional references, Nature Portfolio reporting summaries, source data, extended data, supplementary information, acknowledgements, peer review information; details of author contributions and competing interests; and statements of data and code availability are available at 10.1038/s41586-025-08728-2.

## Supplementary information

Supplementary Information Supplementary Tables 1–6 and references.

Reporting Summary

## Source data

Source Data Figs. 1–4 and Extended Data Figs. 1–10

## Data Availability

The data discussed in this publication have been deposited and are accessible through GEO Series accession number GSE287123. Source data are provided with this paper.

## Code availability

Computational code and relevant files used in this publication are available at Zenodo (10.5281/zenodo.14498318)^57^.
