# Supplementary material for: Regulated somatic hypermutation enhances antibody affinity maturation
Source: Nature. 2025 Mar 19;641(8062):495–502. doi: 10.1038/s41586-025-08728-2 (PMC12058521; doi:10.1038/s41586-025-08728-2)
Supplement: Supplementary file 1 — Supplementary Tables 1–6 and references. [file 41586_2025_8728_MOESM1_ESM.docx]

**Supplementary Information for:**

Regulated somatic hypermutation enhances antibody affinity maturation

Julia Merkenschlager^#,^*^,1,8^, Andrew G.T. Pyo^#,2^, Gabriela S. Silva Santos^1^, Dennis Schaefer-Babajew^1^, Melissa Cipolla^1^, Harald Hartweger^1^, Alexander D. Gitlin^3,4^, Ned S. Wingreen^*,5,6^, and Michel C. Nussenzweig^*,1,7^

^#^Co–first authors

^1^Laboratory of Molecular Immunology, The Rockefeller University, New York, NY 10065, USA

^2^Department of Physics, Princeton University, Princeton, New Jersey, 08544, USA

^3^Immunology Program and Department of Pathology and Laboratory Medicine, Memorial Sloan Kettering Cancer Center, New York, NY, USA

^4^Immunology and Microbial Pathogenesis Graduate Program, Weill Cornell Graduate School, New York, NY

^5^Department of Molecular Biology, Princeton University, Princeton, New Jersey, 08544, USA

^6^Lewis-Sigler Institute for Integrative Genomics, Princeton University, Princeton, New Jersey 08544, USA

^7^Howard Hughes Medical Institute, The Rockefeller University, New York, NY 10065, USA

^8^Laboratory of Lymphocyte collaboration and competition, Harvard Medical School, Boston, MA 02115.

* address correspondence to: [julia_merkenschlager@hms.harvard.edu](mailto:julia_merkenschlager@hms.harvard.edu) , [nussen@rockefeller.edu](mailto:nussen@rockefeller.edu) and [wingreen@princeton.edu](mailto:wingreen@princeton.edu)

**Supplementary Data Inventory**

**Table 1.** Parameters used in the agent-based models.

**Table 2.** Table summarizes the average percentage of nodes (across all mice) as in **Fig.2c.**

**Table 3.** Table summarizes the average number of sequences (across all mice) as in **Fig. 2d.**

**Table 4.** Table summarizes the average percentage of nodes (across all mice) as in **Fig. 3c.**

**Table 5.** Table summarizes the average number of sequences as in **Fig. 3d.**

**Table 6.** Table summarizes the antibodies used in the study.

**Tables**

| **Parameter** | **Description** | **Value** | **Reference** |
| --- | --- | --- | --- |
| $\tau_{B}$ | B cell lifetime in LZ | 8 h | Thomas et al., 2019^1^Meyer-Hermann, Maini & Iber 2006^2^ |
| $\tau_{\mathrm{div}}$ | B cell division time | 5 h | Gitlin et al., 2015^3^ |
| $\tau_{LZ\to DZ}$ | Duration of LZ B cell to DZ B cell differentiation | 2.04 h | Victora et al., 2010^4^ |
| $\tau_{DZ\to LZ}$ | Duration of DZ B cell to LZ B cell differentiation | 5.88 h | Victora et al., 2010^4^ |
| $D_{\max}$ | Maximum number of divisions in the DZ | 6 | Gitlin et al., 2014^5^ |
| $r_{\mathrm{TFH}}$ | T_FH_ cell – B cell interaction rate | 0.4 h^-1^ | Thomas et al., 2019^1^; Meyer-Hermann Maini & Iber 2006^2^ |
| $r_{\mathrm{FDC}}$ | Maximum B cell – FDC interaction rate | 0.75 h^-1^ | N/A |
| $A_{\min}$ | Selection stringency | 2 | N/A |
| $A_{0}$ | Selection stringency scale | 5 | N/A |

**Table 1.** Simulation parameters. Values of parameter used in the simulation with a brief description and references. Parameters determining the selection stringencies were chosen to maintain the total number of B cells in the GC at approximately 3000. The value of maximum B cell – FDC interaction rate $r_{\mathrm{FDC}}$ was chosen such that a B cell expressing BCRs with affinity $a=\left\langle a \right\rangle$ will divide twice on average upon selection by a T_FH_ cell.

**Table 2.** Table summarizes the average percentage of nodes (across all mice) made up of 1, 2-15 or >15 identical sequences, when only considering expanded clones (excluding singles) as in **Fig.2c**.

**Table 3.** Table summarizes the average number of sequences (across all mice) that contributed to nodes containing 1, 2-15 or >15 identical sequences when all cells were considered (i.e. clones and singles) as in **Fig. 2d**

**Table 4.** Table summarizes the average percentage of nodes (across all mice) made up of 1, 2-15 or >15 identical sequences in sum or RBD+ and RBD- compartments, when only considering expanded clones (excluding singles) as in **Fig. 3c.**

**Table 5.** Table summarizes the average number of sequences (across all mice) that contributed to nodes containing 1, 2-15 or >15 identical sequences in the sum, or RBD+ and RBD- compartments when all cells were considered (i.e. clones and singles) as in **Fig. 3d**.

| Antibodies (Name, Clone, Cat/Lot, Company, Dilution) |
| --- |
| anti-mouse CD45.1PE/Cyanine7, Clone A20, Cat: 110729, Biolegend 1/200 |
| anti-mouse CD45.1 FITC, Clone A20, Cat: 11-0453-82, Invitrogen, 1/200 |
| anti-mouse CD45.1 BV421, Clone A20, Lot B376745, Biolegend. 1/200 |
| anti-mouse CD45.1 BV711, Clone A20, Lot B376745, Biolegend. 1/200 |
| anti-mouse CD45.2 FITC, Clone 104, Cat: 553772, BD, 1/ 200 |
| anti-CD45.2 Mouse Monoclonal Antibody PE, Clone 104, Cat 109808, 1/200 |
| anti-mouse CD45.2, Clone 104, Cat 109808, Lot B271929, eBioscience, 1/200 |
| anti-mouse CD45.2, BV421, Clone 104, Cat 109832, Lot B357158, Biolegend ,1/200 |
| anti-mouse CD45.2 APC-Cyanine, clone 104, Cat 109824, Biolegend 1/200 |
| anti-mouse/human PE CD45R/B220, Clone RA3-6B2, Cat 103208, Biolegend 1/200 |
| anti mouse CD45R/B220, Cat 563793, Lot 3135095, BD 1/200 |
| anti mouse CD45R/B220 FITC, Cat 110-0452-85, Invitrogen, 1/200 |
| anti mouse CD45R/B220 BV421, Cat 103240, Biolegend, 1/200 |
| anti mouse CD45R/B220 BUV395, Cat 563793, Lot 3135095, BD, 1/200 |
| anti-mouse CD38, Clone 90/CD38, Cat 553764, BD Bioscience, 1/200 |
| anti-mouse CD38, Clone 90/CD38, Cat 553764, BD Bioscience, 1/200 |
| anti-mouse GL7 PB, Clone GL7, Cat 144614, Biolegend, 1/200 |
| anti-mouse GL7 FITC, Clone GL7, Cat 144603, Biolegend, 1/200 |
| anti-mouse Pecy7 CD95, Clone Jo2, Cat 557653, lot 2145378, BD, 1/200 |
| anti-mouse DEC205R BV421, Clone V18-9449, Cat: 566376,1/200 |
| anti-mouse Igm[a] FITC, Clone: DS-1 Cat 553516, Lot 2026902, BD, 1/200 |
| anti-mouse Ig light chain PE, Clone:RML-41,Cat 407308, Biolegend, 1/200 |
| anti-mouse Ig light chain APC, Clone:RML-41, Cat: 407306, Biolegend, 1/200 |
| anti-mouse CD38 APC, Clone 90Cat 102712, BD Bioscience, 1/200 |
| anti-mouse CD38, Clone 90. Cat 553764, BD Bioscience 1/200 |
| anti-mouse CD38, Clone 90, Cat 102719, Lot B371397, Biolegend 1/200 |
| anti-mouse CD86 APC, Clone GL-1, Cat 4332810, Biolegend 1/200 |
| anti-mouse CXCR4 APC, Clone GL-1, Cat 4332810, Biolegend 1/200 |
| anti-mouse CXCR4 APC, Clone 2B11 Cat 146507 BD Bioscience 1/200 |
| DAP solution, Clone 2B11, Cat 564907, BD Bioscience, 1/200 |
| TotaISeq™--C0301 anti-mouse Hashtag 1 Antibody 155861, Biolegend 1ug |
| TotaISeq™--C0302 anti-mouse Hashtag 2 Antibody 155863, Biolegend 1ug |
| TotalSeq™--C0303 anti-mouse Hashtag 3 Antibody 155865, Biolegend 1ug |
| TotalSeq™--Co304 anti-mouse Hashtag 4 Antibody 155867, Biolegend 1ug |
| TotalSeq™--C0305 anti-mouse Hashtag 5 Antibody, Biolegend 1ug |
| TotaISeq™--C0306 anti-mouse Hashtag 6 Antibody, Biolegend 1ug |
| TotalSeq™--C0307 anti-mouse Hashtag 7 Antibody, Biolegend 1ug |
| TotaISeq™--C0308 anti-mouse Hashtag 8 Antibody, Biolegend 1ug |
| TotalSeq™--C0309 anti-mouse Hashtag 9 Antibody, Biolegend 1ug |
| TotaISeq™--C0310 anti-mouse Hashtag 10 Antibody, Biolegend 1ug |
| TotalSeq™--C0096 anti-mouse CD45 Antibody, Biolegend 1ug |

**Table 6.** Table summarizes the antibodies used in the study.

1 Thomas, M. J., Klein, U., Lygeros, J. & Rodríguez Martínez, M. A Probabilistic Model of the Germinal Center Reaction. *Front Immunol* **10**, 689 (2019). <https://doi.org:10.3389/fimmu.2019.00689>

2 Meyer-Hermann, M. E., Maini, P. K. & Iber, D. An analysis of B cell selection mechanisms in germinal centers. *Math Med Biol* **23**, 255-277 (2006). <https://doi.org:10.1093/imammb/dql012>

3 Gitlin, A. D. *et al.* HUMORAL IMMUNITY. T cell help controls the speed of the cell cycle in germinal center B cells. *Science* **349**, 643-646 (2015). <https://doi.org:10.1126/science.aac4919>

4 Victora, G. D. *et al.* Germinal Center Dynamics Revealed by Multiphoton Microscopy with a Photoactivatable Fluorescent Reporter. *Cell* **143**, 592-605 (2010). <https://doi.org:https://doi.org/10.1016/j.cell.2010.10.032>

5 Gitlin, A. D., Shulman, Z. & Nussenzweig, M. C. Clonal selection in the germinal centre by regulated proliferation and hypermutation. *Nature* **509**, 637-640 (2014). <https://doi.org:10.1038/nature13300>
