# Supplementary material for: Regulated somatic hypermutation enhances antibody affinity maturation
Source: Nature. 2025 Mar 19;641(8062):495–502. doi: 10.1038/s41586-025-08728-2 (PMC12058521; doi:10.1038/s41586-025-08728-2)
Supplement: Supplementary file 2 — Reporting Summary [file 41586_2025_8728_MOESM2_ESM.pdf]

nature portfolio

Corresponding author(s): Michel Nussenzweig

Last updated by author(s): 01/15/52025

Reporting Summary

Nature Portfolio wishes to improve the reproducibility of the work that we publish. This form provides structure for consistency and transparency in reporting. For further information on Nature Portfolio policies, see our Editorial Policies and the Editorial Policy Checklist.

Statistics

For all statistical analyses, confirm that the following items are present in the figure legend, table legend, main text, or Methods section.

n/a	Confirmed	
☐	☒	The exact sample size (*n*) for each experimental group/condition, given as a discrete number and unit of measurement
☐	☒	A statement on whether measurements were taken from distinct samples or whether the same sample was measured repeatedly
☐	☒	The statistical test(s) used AND whether they are one- or two-sided<br>*Only common tests should be described solely by name; describe more complex techniques in the Methods section.*
☐	☒	A description of all covariates tested
☐	☒	A description of any assumptions or corrections, such as tests of normality and adjustment for multiple comparisons
☐	☒	A full description of the statistical parameters including central tendency (e.g. means) or other basic estimates (e.g. regression coefficient) AND variation (e.g. standard deviation) or associated estimates of uncertainty (e.g. confidence intervals)
☐	☒	For null hypothesis testing, the test statistic (e.g. *F*, *t*, *r*) with confidence intervals, effect sizes, degrees of freedom and *P* value noted<br>*Give P values as exact values whenever suitable.*
☐	☒	For Bayesian analysis, information on the choice of priors and Markov chain Monte Carlo settings
☐	☒	For hierarchical and complex designs, identification of the appropriate level for tests and full reporting of outcomes
☐	☒	Estimates of effect sizes (e.g. Cohen's *d*, Pearson's *r*), indicating how they were calculated

Our web collection on statistics for biologists contains articles on many of the points above.

Software and code

Policy information about availability of computer code

Data collection	Flow cytometry data was collected using FACDIVA version 8.0.2.
Data analysis	MacVector was used for sequence analysis. Graph Prism 9 was used for data analysis and for graph generation. In addition, We used cellranger (v3.0.2) from 10X Genomics for single-cell UMI quantification and TCR clonotype assembly. We used Seurat (v3.1.2) an R package to analyze single cell RNA-seq data and to identify differentially expressed genes; graphs were created using R language. For bulk-RNA seq analysis we used kallisto (v.0.46) to map sequence reads to Mus musculus transcriptome (GRCm38/ Ensembl release 99). Kallisto TPM values were converted to absolute counts using tximport (v1.12.3) R package and DESeq2 (v.1.24.0) .

For manuscripts utilizing custom algorithms or software that are central to the research but not yet described in published literature, software must be made available to editors and reviewers. We strongly encourage code deposition in a community repository (e.g. GitHub). See the Nature Portfolio guidelines for submitting code & software for further information.

Data

Policy information about availability of data

All manuscripts must include a data availability statement. This statement should provide the following information, where applicable:

- Accession codes, unique identifiers, or web links for publicly available datasets
- A description of any restrictions on data availability
- For clinical datasets or third party data, please ensure that the statement adheres to our policy

The authors declare that all data supporting the findings of this study are available within the article and its supplementary file or from the corresponding author upon reasonable request. The data discussed in this publication have been deposited Gene Expression Omnibus will be accessible through GEO series accession number GSE287123. All code used is accessible through https://doi.org/10.5281/zenodo.14498318

Research involving human participants, their data, or biological material

Policy information about studies with human participants or human data. See also policy information about sex, gender (identity/presentation), and sexual orientation and race, ethnicity and racism.

Reporting on sex and gender	n/a
Reporting on race, ethnicity, or other socially relevant groupings	n/a
Population characteristics	n/a
Recruitment	n/a
Ethics oversight	n/a

Note that full information on the approval of the study protocol must also be provided in the manuscript.

Field-specific reporting

Please select the one below that is the best fit for your research. If you are not sure, read the appropriate sections before making your selection.

[X] Life sciences [ ] Behavioural & social sciences [ ] Ecological, evolutionary & environmental sciences

For a reference copy of the document with all sections, see nature.com/documents/nr-reporting-summary-flat.pdf

Life sciences study design

All studies must disclose on these points even when the disclosure is negative.

Sample size	Groups of 3-10 mice were used in immunizations. Sample size was determined based on the common standard in the field. n>3 mice/group were used and all the animal experiments were repeated 2-3 times. The number of independent samples used in the experiment are reported in the figure legends.
Data exclusions	We did not exclude any samples. Age and sex matched were used in all experiments. Reported in figure legends.
Replication	Each experiment was performed 2-3 times. A total of 7 mice were used to study NP-OVA elicited GCs. A total of 4 mice were used to compare SARS-CoV-2-RBD elicited GCs. A total of 4 mice were used to compare SARS-COVID-19 mRNA vaccination elicited GCs. A total of 6 mice were used to compare SHM in boosted GC B1-8hi H2Bb-mCherryDEC-205+/+ . Appropriate controls are used in each experiment.n/a
Randomization	Litter mate controls were used for in house strain. Otherwise, C57BL/6 wild type mice were purchased from The Jackson and divided into sex matched and age matched groups
Blinding	Mice were homogenous in sex and age prior to grouping. Investigators were not blinded in this study

Reporting for specific materials, systems and methods

We require information from authors about some types of materials, experimental systems and methods used in many studies. Here, indicate whether each material, system or method listed is relevant to your study. If you are not sure if a list item applies to your research, read the appropriate section before selecting a response.

Materials & experimental systems

n/a	Involved in the study
☐	☒ Antibodies
☒	☐ Eukaryotic cell lines
☒	☐ Palaeontology and archaeology
☐	☒ Animals and other organisms
☒	☐ Clinical data
☒	☐ Dual use research of concern
☒	☐ Plants

Methods

n/a	Involved in the study
☒	☐ ChIP-seq
☐	☒ Flow cytometry
☒	☐ MRI-based neuroimaging

Antibodies

Antibodies used

The updated manuscript includes a complete table detailing all the antibodies used:

Name, Clone, Cat/Lot, Company, Dilution
anti-mouse CD45.1PE/Cyanine7, Clone A20, Cat: 110729, Biolegend 1/200
anti-mouse CD45.1 FITC, Clone A20, Cat: 11-0453-82, Invitrogen, 1/200
anti-mouse CD45.1 BV421, Clone A20, Lot B376745, Biolegend. 1/200
anti-mouse CD45.1 BV711, Clone A20, Lot B376745, Biolegend. 1/200
anti-mouse CD45.2 FITC, Clone 104, Cat: 553772, BD, 1/ 200
anti-CD45.2 Mouse Monoclonal Antibody PE, Clone 104, Cat 109808, 1/200
anti-mouse CD45.2, Clone 104, Cat 109808, Lot B271929, eBioscience, 1/200
anti-mouse CD45.2, BV421, Clone 104, Cat 109832, Lot B357158, Biolegend ,1/200
anti-mouse CD45.2 APC-Cyanine, clone 104, Cat 109824, Lot B335012, Biolegend 1/200
a anti-mouse/human PE CD45R/B220 Antibody, Clone RA3-6B2, Cat 103208, Biolegend 1/200
nti mouse CD45R/B220, Cat 563793, Lot 3135095, BD 1/200
anti mouse CD45R/B220 FITC, Cat 110-0452-85, Invitrogen, 1/200
anti mouse CD45R/B220 BV421, Cat 103240, biolegend, 1/200
anti mouse CD45R/B220 BUV395, Cat 563793, Lot 3135095, BD, 1/200
anti-mouse CD38, Clone 90/CD38, Cat 553764, BD Bioscience, 1/200
anti-mouse CD38, Clone 90/CD38, Cat 553764, BD Bioscience, 1/200
anti-mouse GL7 PB, Clone GL7, Cat 144614, Lot B306510, Biolegend, 1/200
anti-mouse GL7 FITC, Clone GL7, Cat 144603, Biolegend, 1/200
anti-mouse Pecy7 CD95, Clone Jo2, Cat 557653, lot 2145378, BD, 1/200
anti-mouse DEC205R BV421, Clone V18-9449, Cat: 566376, Lot B393190 1/200 dilution
anti-mouse Igm[a] FITC, Clone: DS-1 Cat 553516, Lot 2026902, BD, 1/200
anti-mouse Ig light chain PE, Clone:RML-41,Cat 407308, Lot: B321400, Biolegend, 1/200
anti-mouse Ig light chain APC, Clone:RML-41, Cat: 407306, Lot: B382718, Biolegend, 1/200
anti-mouse CD38 APC, Clone 90Cat 102712, BD Bioscience, 1/200
anti-mouse CD38, Clone 90. Cat 553764, BD Bioscience 1/200
anti-mouse CD38, Clone 90, Cat 102719, Lot B371397, Biolegend 1/200
anti-mouse CD86 APC, Clone GL-1, Cat 4332810, Biolegend 1/200
anti-mouse CXCR4 APC, Clone GL-1, Cat 4332810, Biolegend 1/200
anti-mouse CXCR4 APC, Clone 2B11 Cat 146507 BD Bioscience 1/200
DAP solution Clone 2B11 Cat 564907 BD Bioscience 1/200
TotalSeq™--C0301 anti-mouse Hashtag 1 Antibody 155861, Biolegend 1ug
TotalSeq™--C0302 anti-mouse Hashtag 2 Antibody 155863, Biolegend 1ug
TotalSeq™--c0303 anti-mouse Hashtag 3 Antibody 155865,Biolegend 1ug
TotalSeq™--co304 anti-mouse Hashtag 4 Antibody 155867,Biolegend 1ug
TotalSeq™--c0305 anti-mouse Hashtag 5 Antibody, Biolegend 1ug
TotalSeq™--C0306 anti-mouse Hashtag 6 Antibody , Biolegend 1ug
TotalSeq™--C0307 anti-mouse Hashtag 7 Antibody, Biolegend 1ug
TotalSeq™--C0308 anti-mouse Hashtag 8 Antibody ,Biolegend 1ug
TotalSeq™--C0309 anti-mouse Hashtag 9 Antibody,Biolegend 1ug
TotalSeq™--C0310 anti-mouse Hashtag 10 Antibody, Biolegend 1ug
TotalSeq™--C0096 anti-mouse CD45 Antibody, Biolegend 1ug

Validation

All fluorescent antibodies validated on the manufacturers website.

Animals and other research organisms

Policy information about studies involving animals; ARRIVE guidelines recommended for reporting animal research, and Sex and Gender in Research

Laboratory animals

Mus musculus

C57BL/6, tTa-H2B-mCh mice, B18hi, B18low and B18hi DEC205−/− , mice were generated and maintained at Rockefeller University.

Mice of both sexes, 6-10 Weeks of age.

Wild animals

no wild animal

Reporting on sex

both sexes where used

Field-collected samples

n/a

Ethics oversight

All procedures in mice were performed in accordance to protocols approved by the Rockfeller University IACUC. All animal experiments were performed according to the protocols approved by the Institutional Animal Care and Use Commitee of NIAID, NIH

Note that full information on the approval of the study protocol must also be provided in the manuscript.

Plants

Seed stocks

Report on the source of all seed stocks or other plant material used. If applicable, state the seed stock centre and catalogue number. If plant specimens were collected from the field, describe the collection location, date and sampling procedures.

Novel plant genotypes

Describe the methods by which all novel plant genotypes were produced. This includes those generated by transgenic approaches, gene editing, chemical/radiation-based mutagenesis and hybridization. For transgenic lines, describe the transformation method, the number of independent lines analyzed and the generation upon which experiments were performed. For gene-edited lines, describe the editor used, the endogenous sequence targeted for editing, the targeting guide RNA sequence (if applicable) and how the editor was applied.

Authentication

Describe any authentication procedures for each seed stock used or novel genotype generated. Describe any experiments used to assess the effect of a mutation and, where applicable, how potential secondary effects (e.g. second site T-DNA insertions, mosiacism, off-target gene editing) were examined.

Flow Cytometry

Plots

Confirm that:

- [ ] The axis labels state the marker and fluorochrome used (e.g. CD4-FITC).
- [x] The axis scales are clearly visible. Include numbers along axes only for bottom left plot of group (a 'group' is an analysis of identical markers).
- [ ] All plots are contour plots with outliers or pseudocolor plots.
- [x] A numerical value for number of cells or percentage (with statistics) is provided.

Methodology

Sample preparation

Single cell suspensions were obtained from popiteal lymph nodes or spleens of experimental mice, T cells and B cells were isolated by negative selection using PE-Easy Sep selection. Otherwise untouched single cell suspensions were stained for analysis.

Instrument

BD FACSSYMPHONY

Software

FlowJo version v10.4.2

Cell population abundance

purity was above 95%

Gating strategy

Samples were indexed with TotalSeqC (BioLegend) cell surface antibodies and live, lineage-, B220+, GL7+, Fas+, mCherryhigh and mCherrylow GC cells were purified by flow cytometry and loaded onto a Chromium Controller (10x Genomics).

For single cell RNA sequencing of RBD challenged mice, single cell suspensions were prepared from draining lymph nodes on day 14 after immunization. Samples were indexed with TotalSeqC (BioLegend) cell surface antibodies and live, lineage-, B220 +, CD38-, Fas+, and RBD- and RBD+ GC cells were purified by flow cytometry and loaded onto a Chromium Controller (10x Genomics).

- [x] Tick this box to confirm that a figure exemplifying the gating strategy is provided in the Supplementary Information.
